# Transient viral replication during analytical treatment interruptions in SIV infected macaques can alter the rebound-competent viral reservoir

**DOI:** 10.1371/journal.ppat.1009686

**Published:** 2021-06-18

**Authors:** Taina T. Immonen, Christine M. Fennessey, Leslie Lipkey, Abigail Thorpe, Gregory Q. Del Prete, Jeffrey D. Lifson, Miles P. Davenport, Brandon F. Keele

**Affiliations:** 1 AIDS and Cancer Virus Program, Frederick National Laboratory, Frederick, Maryland, United States of America; 2 Infection Analytics Program, Kirby Institute for Infection and Immunity, University of New South Wales, Sydney, New South Wales, Australia; University of Pennsylvania, UNITED STATES

## Abstract

Analytical treatment interruptions (ATIs) of antiretroviral therapy (ART) play a central role in evaluating the efficacy of HIV-1 treatment strategies targeting virus that persists despite ART. However, it remains unclear if ATIs alter the rebound-competent viral reservoir (RCVR), the virus population that persists during ART and from which viral recrudescence originates after ART discontinuation. To assess the impact of ATIs on the RCVR, we used a barcode sequence tagged SIV to track individual viral lineages through a series of ATIs in Rhesus macaques. We demonstrate that transient replication of individual rebounding lineages during an ATI can lead to their enrichment in the RCVR, increasing their probability of reactivating again after treatment discontinuation. These data establish that the RCVR can be altered by uncontrolled replication during ATI.

## Author summary

While HIV-1 replication can be effectively controlled by combination antiretroviral therapy in most individuals, it does not clear long-lived viral reservoirs that were established before ART was started. This necessitates life-long adherence to therapy to prevent re-emergence of infection. Clinical trials evaluating interventions that would allow people to safely stop ART rely on treatment interruptions to determine if, and when, viral rebound occurs. However, it remains unclear if extended viral replication during treatment interruptions can increase the size of the viral reservoir or alter its genetic composition. To directly assess the impact of replication on the viral reservoir, we used a barcoded simian immunodeficiency virus in nonhuman primates to track the reactivation and replication dynamics of individual viral lineages across a series of treatment interruptions. We found that transient replication of rebounding viral variants during an interruption increased their probability of reactivating again when treatment was subsequently discontinued. Our findings suggest that the viral reservoir can become enriched by viral lineages that are actively replicating during treatment interruptions, which could have important implications for clinical trial participants.

## Introduction

Despite successful suppression of virus replication with antiretroviral therapy (ART), HIV-1 persists and can rekindle ongoing virus replication when treatment is discontinued [1–4]. This rebound competent viral reservoir (RCVR) is dominated by resting CD4+ T cells, including expanded CD4+ T cell clones harboring clonally integrated proviruses, that do not express viral antigens, and thus go unrecognized by host immune responses [5–8]. Life-long suppressive ART is required to prevent the recrudescence of systemic virus replication from the RCVR. A large-scale international research effort is focused on developing treatment strategies that would allow people to safely stop ART, either through eradicating the RCVR or by augmenting host immune responses to achieve sustained control of virus replication following ART discontinuation [9–11]. However, because validated biomarkers predictive of RCVR reduction or post-treatment viral control are not yet available, clinical trials evaluating such interventions rely on analytical treatment interruptions (ATIs) to assess time to viral recrudescence and/or rebound setpoint viremia as surrogate measures of RCVR size and virologic control [12–15]. This raises questions about potential risks of viral replication during ATIs for study participants and possible changes in the size or composition of the RCVR.

While short-term ATIs are generally considered safe for humans infected with HIV [13–15], concerns remain about whether viral replication after treatment discontinuation can alter the composition of the RCVR through enrichment of the specific viral genomes that rebound. Evidence thus far indicates that brief ATIs of 4–6 weeks do not meaningfully increase the absolute size of the RCVR based on comparison of pre-ATI and post-ATI surrogate measures of RCVR size, including total HIV-1 vDNA concentrations, cell-associated viral RNA, and frequency of resting CD4+ T cells harboring inducible replication-competent virus as assessed using quantitative viral growth assays (QVOA) [16–19]. However, these *ex vivo* assays do not directly assess the size of the persisting vDNA population that can lead to recrudescent viremia after ART interruption, and are not in general predictive of time-to-rebound [19–22], which is considered the most relevant measure of the size of the RCVR. Total HIV-1 vDNA levels based on viral gag gene quantification are difficult to interpret because they are typically dominated by defective proviruses that do not contribute to viral rebound, particularly if ART is initiated during the chronic phase of HIV-1 infection, and therefore tend to greatly overestimate the size of the RCVR [8,23,24]. On the other hand, QVOA tends to underestimate the size of the RCVR because not all proviruses are induced to reactivate after a single round of *ex vivo* stimulation, and reactivation in culture may differ from reactivation *in vivo* [8]. In addition to their potentially limited relevance for the RCVR, these assays may not be able to detect small but potentially meaningful changes due to their limited sample size, dynamic range and precision [1,8,25–27].

Even if the absolute size of the RCVR does not measurably increase during ATIs, it may become enriched with rebounding lineages that replicate during the ATI. Because the only way to directly characterize the RCVR is to sample the rebounding lineages that emerge during an ATI, it is challenging to assess changes to its genetic composition. A recent study evaluating the effect of short-term ATIs on the viral reservoir did not find any difference in the genetic composition of QVOA and HIV-1 vDNA populations before or after ATI [18]. However, in line with several other studies [28–32], rebound viruses were exceedingly rare among the QVOA and HIV-1 vDNA virus populations sampled prior to ART discontinuation. Interestingly, rebounding lineages were frequently related to pre-ATI sequences through recombination [28,30,31], suggesting that the pre-ATI virus population was not sampled deeply enough or alternatively, that rebound viremia was sampled too late to detect the initially rebounding viruses [33]. Overall, the lack of concordance between rebounding lineages and the virus populations sampled prior to the ATIs highlight the difficulty in characterizing the potentially large, diverse and heterogeneously distributed RCVR.

To overcome challenges posed by indirect and limited sampling of the RCVR and to allow for interventions not feasible in a clinical setting, we used a nonhuman primate (NHP) model to assess if transient replication of rebounding lineages during ATIs can alter the RCVR. We used the genetically barcoded but otherwise isogenic virus SIVmac239M [26,34–37] to track individual barcoded viral lineages through multiple cycles of ART suppression and interruption. The 34-nt genetic tag can be deeply sequenced using next generation sequencing (NGS) methods to estimate the relative proportion of each barcode [26,35]. Importantly, we have previously shown that the barcode remains intact during several months of replication and have not found any indication that different variants, which are isogenic except for the barcode sequences, differ in their replicative capacity [26]. The ability to quantify the replication of thousands of individual clonal variants greatly increases analytic resolution into the structure of the virus population, allowing detection of nuanced changes to its composition [34], and provides a way to directly estimate the rate of reactivation from latency for individual animals [26,38].

In this study, the artificial genetic diversity present in the virus stock allowed for rapid seeding with a diverse population of viral barcode lineages, enabling early ART initiation and thereby preventing the accumulation of immune escape mutations and other phenotypic differences in the RCVR that could potentially confound the dynamics of rebound [39]. To assess the contribution of viral replication to the RCVR, we precisely measured both the quantity and genetic make-up of the distinct viral variants comprising the replicating virus population before ART initiation and during successive ATIs. This allowed us to directly quantify the impact of transient replication of rebounding viral lineages during an ATI on the likelihood of their reactivating again during subsequent ATIs. Replication of rebounding lineages during ATI was associated with increased likelihood of reactivating again when ART was subsequently discontinued, proportionate to their level of replication during the prior ATI, demonstrating that transient replication of rebounding lineages during ATI reseeded the RCVR. Viral barcode lineages that were predominant in ATI rebound viremia were also enriched in PBMC SIV cell-associated vDNA (CA-vDNA) measured after ART re-initiation and viral suppression, consistent with their increased contribution to the RCVR. Overall, our findings suggest that the potential for reseeding of the RCVR with viral lineages that are actively replicating to higher levels during an ATI may be an important consideration for future treatment decisions.

## Results

### Viral load kinetics during animal study

**Fig 1:**
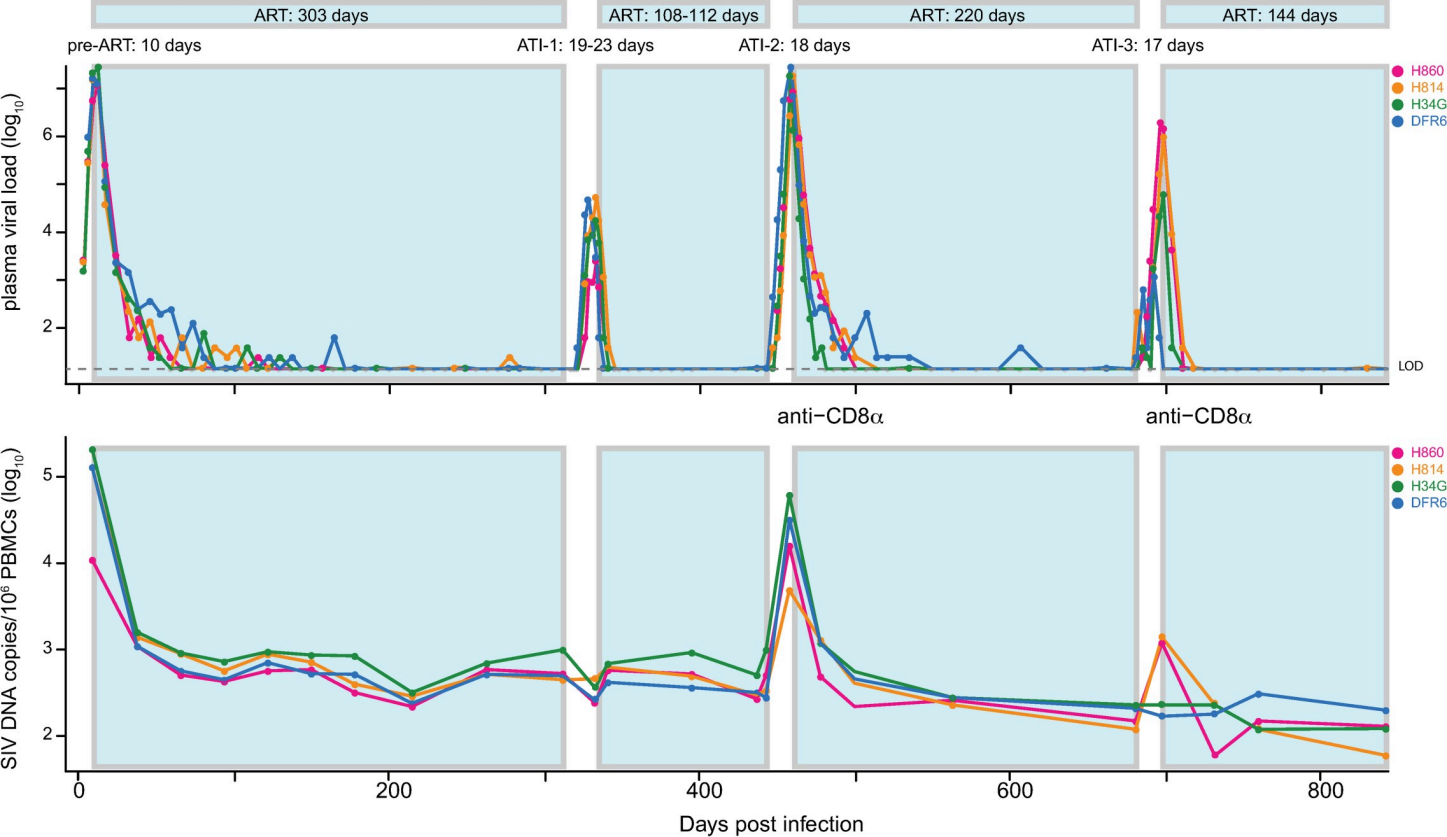
Viral replication dynamics in plasma and PBMC CA-DNA. Four Indian-origin Rhesus macaques were infected with SIVmac239M on day 0 and treated with ART on day 10. Viral RNA (copies per mL) were measured in plasma over 843 days with values below 15 copies/mL shown in grey. The animals underwent three treatment interruptions, between 313 to 336 dpi, 444 to 462 dpi, and 682 to 699 dpi. CD8 depletion with anti-CD8α mAb was performed 3 days prior to the last two ATIs. CA-vDNA (per 10^6^ PBMC) was also quantified via qPCR at various times post infection.

To assess whether transient viral replication after treatment discontinuation can alter the RCVR, we used a barcoded SIV [26] in rhesus macaques to track replication of distinct viral lineages across a series of ATIs. Four rhesus macaques (H860, H814, H34G and DFR6) were infected intravenously with SIVmac239M followed by daily combination ART administration (TDF/FTC/DTG) starting at 10 days post-infection (dpi) (Fig 1). A total of 3 treatment interruptions of 11 to 21 days were performed after periods of sustained viral suppression at 313 dpi, 444 dpi, and 682 dpi, respectively. The latter two ATIs were preceded by experimental administration of a depleting anti-CD8α antibody 3 days prior to ART withdrawal. Plasma viral load (pVL) and PBMC SIV CA-vDNA (Fig 1) were measured at multiple times throughout study and viral sequences were assessed longitudinally during both suppressive ART phases and during ATIs.

### Representation of pre-ART plasma virus lineages in PBMC viral DNA during ART

SIV CA-vDNA sampled during ART has frequently been used as a surrogate marker for the RCVR. To better understand the dynamics underlying the establishment of the persistent viral DNA population, we assessed its relatedness to plasma virus replicating before ART was initiated. To characterize the replicating virus and cell-associated viral DNA populations, we used next generation sequencing to identify the number of distinct viral barcodes and their relative abundance in peak pre-ART plasma (10 dpi) and in CA-vDNA sampled during suppressive ART between 172 dpi to 313 dpi. Barcode sequencing revealed a large number of distinct lineages in the pre-ART plasma of each animal (mean: 588, range: 558–629).

**Fig 2:**
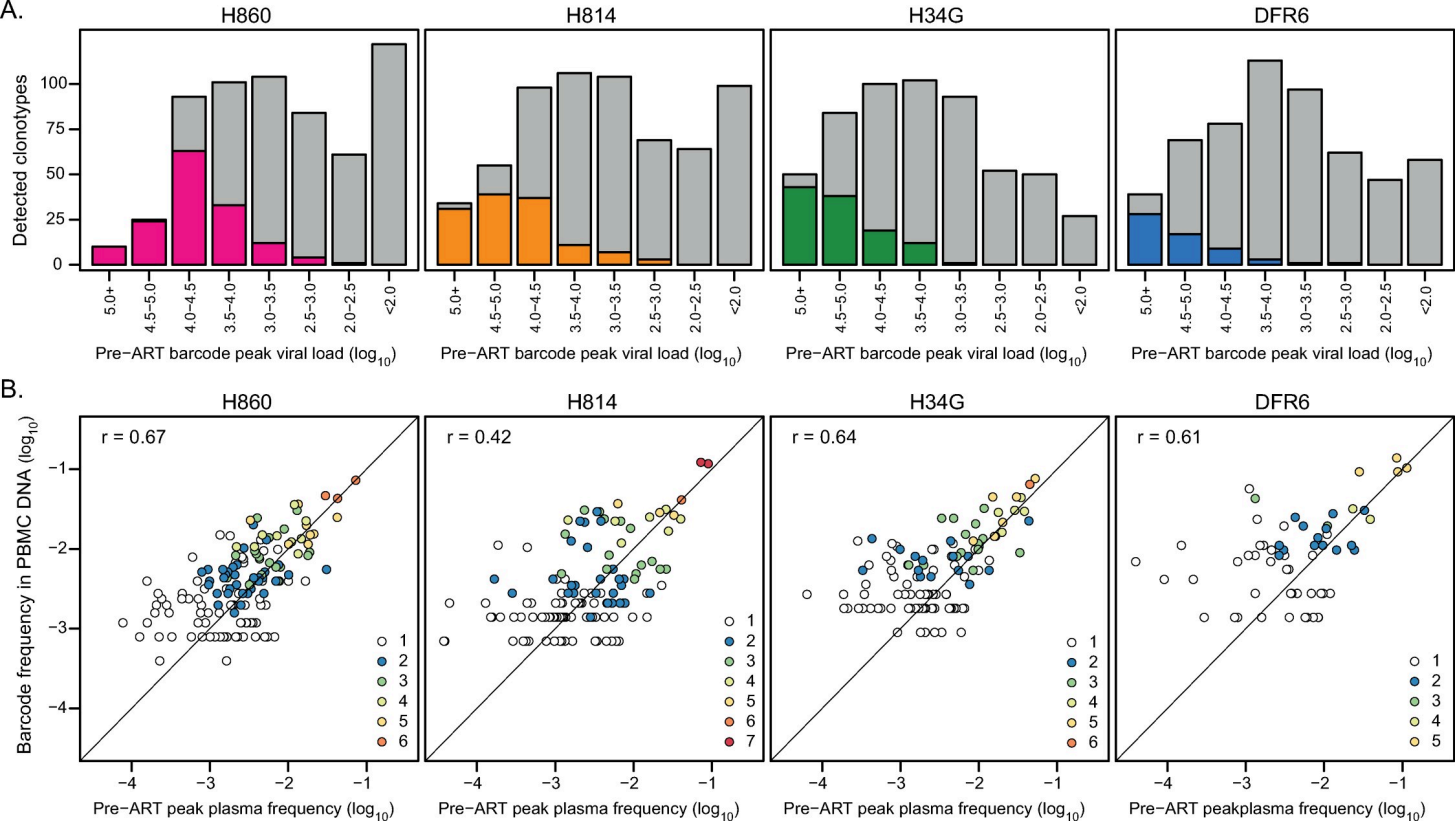
Barcode size in pre-therapy plasma is predictive of detection and relative abundance in PBMC CA-vDNA. (A) barcodes were partitioned into 0.5-log_10_ intervals based on their pre-ART peak plasma SIV RNA viral loads. The grey bars depict the number of barcodes in each viral load category, and the colored bars highlight the number of lineages that were observed in CA-vDNA in at least one PBMC sample during suppressed viremia between 172 and 313 dpi. (B) The pre-ART plasma frequency attributable to each lineage is plotted against its estimated frequency in vDNA. The Pearson correlation between all lineages detected in at least two PBMC samples is indicated for each animal, with the linear regression line shown in black. The dashed grey line represents a theoretical one-to-one ratio of barcode proportions. The color of the points indicates the number of PBMC samples each barcode was detected in. The number of PBMC samples analyzed was 6 for H860, 8 for H814, 6 for H34G and 5 for DFR6. For H814, no single barcode was detected in all 8 samples.

Parallel quantitative assessment of the total vRNA or vDNA level and the proportion of individual barcode sequences allows determination of the contribution of individual viral variants to the total virus present in the specimen. To assess if the viral DNA population that persists during ART is established proportionately to the extent of replication of distinct viral lineages at time of ART initiation, we determined the relationship between the replication of distinct viral barcodes pre-ART and their subsequent detection in CA-vDNA during ART (Fig 2). While nearly all individual barcodes that contributed to pre-ART peak plasma vRNA at levels exceeding 10^5^ copies/mL were detectable as vDNA in PBMC on ART, the likelihood of a barcode being sampled quickly declined as a function of decreasing pre-ART barcode size (with only 0.9% of lineages detected on average for pVL <10^3^)(Fig 2A). Overall, only a small fraction of pre-ART barcodes (4% to 12%) were detected in CA-vDNA of at least one PBMC sample, reflecting the limited depth of sampling possible during ART. To further assess the relationship between the composition of the on-ART PBMC vDNA virus population and the pre-ART plasma virus pool, we compared the relative frequencies of individual barcoded lineages in the two populations (Fig 2B). Relative frequencies of barcodes were significantly correlated between pre-ART plasma and on-ART PBMC CA-vDNA in each animal (mean r = 0.59; range = 0.42 to 0.68; Pearson correlation coefficient; p-value < 0.002 for all animals). The most abundant barcodes, which were generally detected in multiple different PBMC samples, exhibited nearly equivalent plasma and CA-vDNA frequencies. However, the estimated average frequencies of minor pre-ART barcodes were generally measured as higher in PBMC DNA than in plasma, which is likely artificially enriched due to the limited sampling depth of SIV+ cells in PBMCs taken during ART. Together, these results demonstrate that while it is challenging to directly characterize the viral DNA population due to limited sampling during ART, the vDNA pool in PBMC largely reflects the pre-ART replicating virus population present in plasma at the time of ART initiation, even after 10 months of suppressive ART.

### Reactivating lineages are predominant in pre-ART plasma

**Fig 3:**
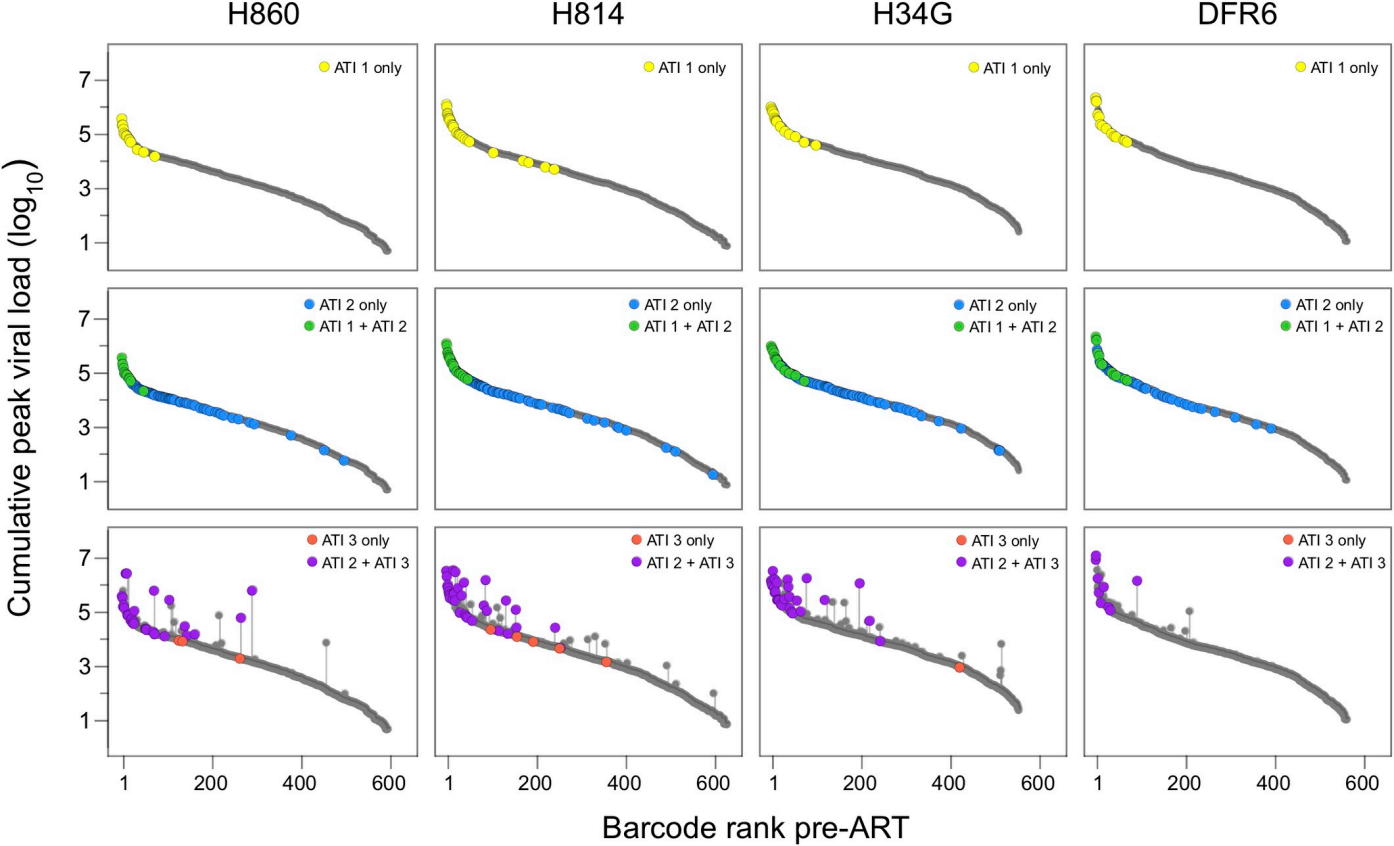
Reactivation of variants based on viral replication. Each row depicts reactivation of lineages in the first (top panels), second (middle panels), or third (bottom panels) treatment interruption. The grey points depict the cumulative peak viral loads (pre-ART plus any previous ATI) attributable to each variant and rank-ordered based on their relative frequency at d10. The dark grey lines depict the pre-ART barcode viral loads and the vertical grey lines correspond to the contribution of replication during ATIs to cumulative barcode viral loads. Barcodes detected at ATI-1 are highlighted in yellow; green if also found in ATI-1; and blue if unique to ATI-2. Barcodes detected during ATI-3 are purple if also detected in ATI-2 and red if unique to ATI-3.

One of the major difficulties in assessing the genetic composition of the RCVR is that it cannot be unambiguously sampled directly during ART and viral sequences isolated from pre-ATI QVOA and viral DNA populations are in general not representative of the lineages that rebound after treatment discontinuation [18,28,30,31]. As described above, the limited depth of sampling during ART and the large absolute size of the persistent virus pool likely contribute to this discrepancy. Because pre-ART plasma correlates to CA-vDNA but with much greater depth of sampling, we next examined if the replicating virus population in plasma prior to ART initiation is predictive of which viral lineages rebound after ART is discontinued. To this end, ART was stopped at 313 dpi and plasma viremia rebound dynamics were assessed by total plasma SIV RNA levels and sequencing. All animals rebounded within 14 days, reaching peak viral loads of 2.6x10^3^–5.5x10^5^ copies/mL (Fig 1). Sequencing the viral barcodes revealed numerous distinct variants at peak rebound viremia (mean = 19, range = 14 to 26), corresponding to an estimated average reactivation rate of 4.0 (sd = 1.4) reactivation events per day (Table 1) [26]. In each animal, all rebounding lineages were detected in pre-ART plasma, with the vast majority (79% to 93%) originating from the top 10% of the pre-ART barcode frequency distribution (Fig 3, top panels). The majority (57% - 86%) of rebounding lineages were also detected in CA-vDNA sampled in PBMCs from each animal prior to ATI-1, consistent with predominant lineages in the pre-ART plasma virus population also being detectable in the viral DNA population. These data indicate that the most abundant variants prior to ART were more likely to reactivate, presumably because they constituted a larger fraction of the total RCVR. We next assessed whether the level of replication of the rebounding lineages pre-ART was also predictive of their contribution to rebound viremia during ATI-1 (S1 Fig). Individual lineage viral loads were significantly correlated between pre-ART and ATI-1 plasma viremia for two of the animals (H860: r = 0.64, p = 0.01; H814: r = 0.79, p = 2E-6; Pearson correlation) but were not in the other two animals (H34G: r = 0.43, p = 0.1, DFR6: r = 0.44, p = 0.1), likely due to the limited number of rebounding lineages. Overall, these results demonstrate that the genetic composition of the replicating virus population prior to ART initiation is predictive of which lineages are most likely to rebound upon ATI and may also influence the relative contributions of the reactivating variants to rebound viremia. Furthermore, when ART is initiated early and there is sufficient genetic variability to discriminate between distinct lineages, the majority of rebounding lineages can be detected in PBMC CA-vDNA sampled prior to treatment discontinuation.

**Table 1:** Summary of viral replication and reactivation kinetics.

	H860	H814	H34G	DFR6
**Viral load (copies/mL)**				
Pre-ART peak	12,000,000	16,000,000	26,000,000	17,000,000
Post ATI 1 Peak	2,600	55,000	18,000	49,000
Post ATI-2 Peak	8,800,000	19,000,000	19,000,000	29,000,000
Post ATI 3 Peak	2,000,000	1,000,000	63,000	1,200
**Number of detectable barcodes**				
Pre-ART peak	596	629	555	563
Post ATI 1 Peak	14	26	16	14
Post ATI-2 Peak	106	116	128	85
Post ATI 3 Peak	31	38	31	12
**Reactivation Rate (per day)**				
Post ATI 1	4.7	5.4	3.2	2.3
Post ATI-2	13.4	14.8	17.3	11.6
Post ATi 3	4.5	5.6	9.0	1.9

### Limited viral replication during an ATI has no detectable impact on SIV CA-vDNA

Concerns about potential adverse effects of ATIs in HIV-infected individuals have been mitigated by findings that HIV-1 total vDNA levels do not significantly increase during short term ATI [16–19,40]. We assessed if the viral DNA population in PBMC was altered in size or composition by limited viral rebound during the initial ATI. Total SIV CA-vDNA levels did not increase after ATI-1 with an average of 628 copies/million PBMC at time of ART discontinuation at 313 dpi and an average of 580 copies/million PBMC cells shortly after re-initiation of ART at 342 dpi (Fig 1). The lack of measurable contribution of this ATI to total SIV vDNA levels is consistent with published clinical data [16–18] and is perhaps not surprising given the over 100-fold lower levels of viral replication that were reached during this ATI compared to pre-ART, as assessed by pVL. We next investigated whether the barcodes that rebounded during ATI-1 were subsequently enriched in the PBMC DNA population. Logistic regression was used to assess if reactivation of particular lineages during ATI-1 increased their likelihood of being detected in post-ATI 1 CA-vDNA (between 346 and 443 dpi), after accounting for their pre-ART level of replication. In all animals, pre-ART peak plasma viremia of individual barcodes was the only significant predictor for detection in PBMC vDNA after ATI and reinstitution of ART (Table 2). These data reflect the limited replication of individual variants during ATI-1 relative to pre-ART (when the RCVR was first established) and indicate that reactivation of distinct viral lineages in and of itself did not lead to their enrichment in CA-vDNA. Overall, modest viral replication during ATI-1 did not measurably alter the amount or genetic composition of vDNA in PBMC, consistent with findings in people undergoing short-term ATIs with relatively low rebound viral loads [16–18]. However, the limited potential for replication-induced reseeding of the RCVR during ATI-1 precluded assessing whether replication of rebounding viral lineages at higher levels, as might be expected during longer-term ATIs in people, can reseed the persistent viral DNA population and its rebound-competent subset.

**Table 2:** Summary of estimated model parameters.

^**1**^ **(A) Predictors of CA-DNA after ATI-1:** **Pre-ART peak VL model**	Est (P-value)	Est (P-value)	Est (P-value)	Est (P-value)
Intercept	-12.3 (<2E-16)	-14.2 (<2E-16)	-13.9 (<2E-16)	-15.5 (<2E-16)
Pre-ART VL (log10)	2.8 (<2E-16)	3.0 (<2E-16)	2.8 (<2E-16)	3.0 (1.5E-14)
AIC	***304***	***253***	***266***	***183***
**Pre-ART peak VL + ATI-1 reactivation model**				
Intercept	-12.1(<2E-16)	-14.4 (<2E-16)	-13.8 (<2E-16)	-14.9 (4E-16)
Pre-ART VL (log10)	2.8 (<2E-16)	3.0 (<2E-16)	2.7 (<2E-16)	2.9 (7E-13)
Reactivation in ATI-1 (yes/no)	0.5 (0.6)	-0.1 (0.8)	0.2 (0.8)	1.3 (0.08)
AIC	***306***	***255***	***268***	***182***
^**2**^ **(B) Predictors of ca-DNA after ATI-2:** **Pre-ART AUC of virus model**				
Intercept	-13.2 (<2E-16)	-14.0 (<2E-16)	-16.0 (<2E-16)	-20.0 (5E-12)
Pre-ART VL (log10)	2.6 (1E-15)	2.8 (<2E-16)	3.0 (<2E-16)	3.7 (9E-11)
AIC	***258***	***269***	***235***	***124***
**Cumulative (pre-ART + ATI-1 + ATI-2) AUC of virus model**				
Intercept	-13.1 (<2E-16)	-14.3 (<2E-16)	-14.6 (<2E-16)	-18.5 (4E-12)
Total VL (log10)	2.6 (1E-15)	2.8 (<2E-16)	2.7 (<2E-16)	3.73(1E-10)
AIC	***225***	***246***	***220***	***112***
^**3**^ **(C) Predictors of reactivation in ATI-3:** **Pre-ART AUC of virus model**				
Probability of reactivation	4.0E-6 (1E-5)	2.3E-6 (4E-5)	1.3E-6 (2E-3)	5.5E-7 (0.2)
AIC	***167***	***185***	***143***	***61***
**Cumulative (pre-ART + ATI-1 + ATI-2) AUC of virus (reseeding) model**				
Probability of reactivation	2.4E-6 (2E-4)	1.7 E-6 (6E-4)	6.8E-7 (0.05)	2.6E-7 (0.4)
AIC	***139***	***152***	***109***	***54***
**Pre-ART AUC of virus + weighted effect of ATI-1 and ATI-2 AUC virus** **(weighted reseeding) model**				
Probability of reactivation	2.7E-6 (1E-4)	1.5E-6 (2E-3)	4.9E-7 (0.1)	3.0E-7 (0.4)
Weight of ATI VL	0.7 (<2E-16)	1.8 (NA)	1.7 (NA)	0.5 (NA)
AIC	***141***	***149***	***110***	***55***

^1^ Logistic regression models for detection of individual barcodes in CA-DNA after ATI-1 (at any time point between 346 and 443 dpi). The sole explanatory variable for the baseline model is the viral load attributable to each barcode at pre-ART peak viremia; the alternative model also includes a binary indicator variable for whether each barcode reactivated during ATI-1.

^2^ Logistic regression models for detection of individual barcodes in CA-DNA after ATI-2 (at any time point between 552 and 682 dpi). The explanatory variable for the baseline model is the AUC of virus attributable to each barcode pre-ART; the explanatory variable for the alternative model is the barcode-specific cumulative AUC of virus (during pre-ART, ATI-1 and ATI-2).

^3^ Binomial models for the probability of reactivation of barcodes in ATI-3 based on their past replication. In the pre-ART and reseeding models, the number of trials corresponds to the pre-ART barcode-specific AUC of virus load (virus copies/mL) and to the cumulative barcode-specific AUC of virus load (during pre-ART, ATI-1 and ATI-2), respectively. In the weighted reseeding model, the number of trials is the sum of the AUC of virus pre-ART and the AUC of virus during ATIs, the latter term weighted by a nonnegative factor to allow for differential contribution of replication to the RCVR during ATIs.

### Depletion of CD8α+ cells increases viral replication and reactivation

To assess whether reseeding can be detected if rebounding lineages replicate to higher levels, we adopted an experimental strategy to increase the overall level of replication during subsequent ATIs. While delaying the re-initiation of ART is expected to increase the plasma viremia AUC during an ATI, prolonged viral replication may lead to the emergence of immune escape mutations and fitness differences between variants thereby complicating the analysis. To circumvent the potentially confounding effects of selection at the time of rebound, we sought to increase virus replication during a short period of time via antibody-mediated *in vivo* depletion of CD8^+^ T lymphocytes. Animals received a depleting anti-CD8α monoclonal antibody (MT807R1) 3 days prior to a second ATI at 444 dpi (ATI-2). Viral rebound was detectable in plasma within 7 days of ART interruption in all animals, with peak plasma viral loads (8.8x10^6^–2.9x10^7^ copies/mL) higher than in ATI-1 and comparable to levels measured during primary infection. We also observed a substantial increase in both the number of rebounding barcodes (mean: 109, range: 84–130) and the estimated average reactivation rate (mean: 14.3 per day; sd = 3.0) relative to ATI-1 (mean number of barcodes: 19; mean average reactivation rate: 4.0 per day)(Table 1). This may be due to known activation of CD4+ T cells following depletion with this antibody [41,42]. The rebounding barcodes spanned the entire pre-ART distribution (Fig 3 middle panels), indicating that even minor lineages found in pre-ART plasma had seeded the RCVR and were capable of reactivating and replicating to detectable levels. Importantly, less than 1% (4 out of 439) of the total variants that reactivated during ATI-2 were not detected in pre-ART plasma. However, only 13% - 23% of the variants that reactivated during ATI-2 were detected at any time in PBMC while on-ART, highlighting the challenges in predicting which lineages reactivate given the limited feasible sampling of viral DNA.

### Pre-ART barcode proportion predicts reactivation and relative contribution to rebound viremia

**Fig 4:**
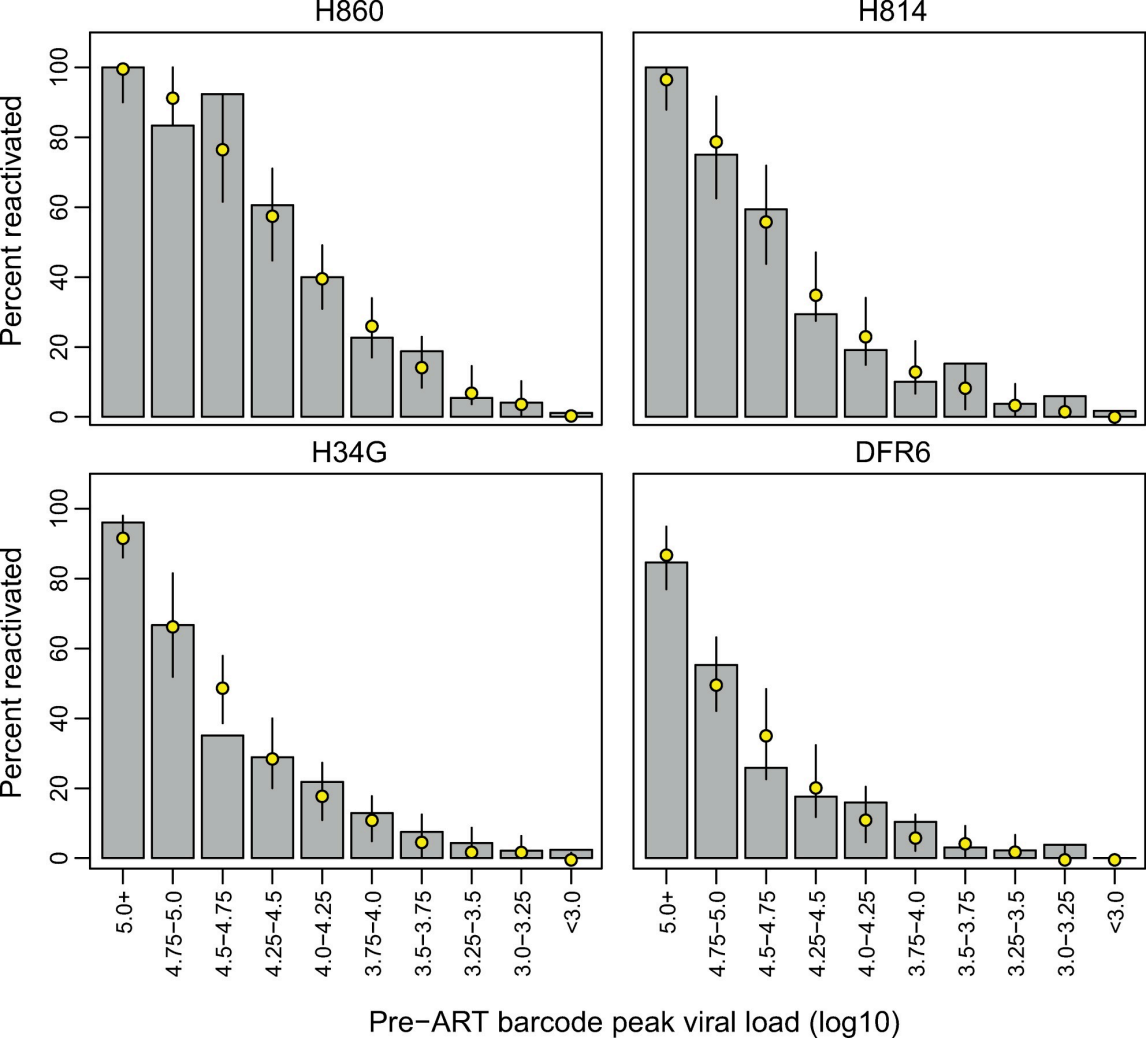
Size of viral lineage pre-ART predicts reactivation after treatment discontinuation. Barcodes were partitioned into 0.25-log_10_ intervals based on their pre-ART plasma viral loads. The grey bars depict the proportion of barcodes from each viral load category that were observed at peak rebound viremia during ATI-2. The yellow points correspond to the median simulated number of reactivated barcodes in each category, with 90% credibility intervals.

The large number of lineages rebounding during ATI-2 allowed us to directly investigate the extent to which the composition of the RCVR reflected that of the replicating virus population at the time of ART initiation. We partitioned the individual rebounding lineages into intervals based on their pre-ART peak viral loads (at 10 dpi) and compared the observed and expected proportions of barcodes that reactivated in each interval (Fig 4). Overall, the data were consistent with the probability of reactivation being directly proportional to the relative frequency of a lineage in pre-ART plasma viremia (Chi-Square goodness-of-fit test, p-values between 0.15 to 0.99), indicating that the RCVR continued to reflect the virus population replicating at the time of ART initiation over 14 months earlier. We further assessed whether the relative abundance of the rebounding variants pre-ART was also predictive of their level of replication during ATI-2. In all animals, the relative contribution of reactivating lineages to ATI-2 rebound plasma viremia was moderately correlated with their pre-ART plasma viral loads (mean r = 0.45; range = 0.24 to 0.64; Pearson correlation coefficient; p-value ≤ 0.01 for all animals), corroborating the trend observed during ATI-1. However, several lineages in each animal replicated substantially better during ATI-2 than during primary infection (S1 Fig). Because the barcoded virus was designed to minimize phenotypic differences between variants, the increase in relative proportion of an individual lineage to rebound viremia is likely determined by the timing of reactivation of that lineage, in relation to the local cellular and/or tissue milieu, rather than a phenotypic difference between barcodes. Taken together, these data indicate that while lineages that are dominant in pre-ART plasma were more likely to reactivate during subsequent ATIs and contribute more to rebound viremia than less abundant variants, even minor lineages can reactivate and replicate substantially, potentially leading to their enrichment in the RCVR.

### Lineage specific replication during ATI can lead to increases in CA-vDNA

While limited viral replication during ATI-1 did not have a measurable impact on PBMC vDNA levels following post-ATI re-suppression (confirming that short ATIs with rapid return to therapy should not materially impact the RCVR), the SIV CA-vDNA levels reached during ATI-2 were over 100-fold higher (Fig 1), substantially increasing the potential of ATI-2 to alter the viral population. To determine if this greater replication resulted in a measurable alteration of the RCVR, we assessed the total SIV CA-vDNA in PBMC following ATI-2. Remarkably, despite initial large increases in CA-vDNA coinciding with viral replication off therapy, total CA-vDNA levels rapidly declined to levels observed prior to ATI-2 after approximately 3 months of reinstituted ART (Fig 1, bottom panel). This implies that the increase in total PBMC DNA during the ATI was predominately in short-lived, infected cells. In contrast to ATI-1, where the plasma SIV levels reached by rebounding variants were less than 1% of their pre-ART viral loads, and therefore had limited potential to reseed the viral CA-DNA population, during ATI-2 we observed several lineages in each animal that replicated as well or better during the second rebound as during primary infection. We thus investigated if substantial transient replication of individual barcoded lineages during ATI-2 led to their enrichment in the post-ATI viral CA-DNA population. We used logistic regression to assess if replication of rebounding variants during ATI-1 and ATI-2 increased their likelihood of being subsequently detected in PBMC SIV CA-DNA during the post-ATI-2 period of re-initiated ART suppression (between 552 and 682 dpi). To estimate the total replication of individual variants pre-ART and during each ATI, we assumed that barcode proportions, assessed at peak viremia, either pre-ART or during the ATI, were constant throughout each off-ART cycle, and determined the proportional contribution of each lineage to the area under the curve (AUC) of plasma viral load. Detection of barcodes in vDNA was explained significantly better when accounting for their cumulative replication estimated in this fashion rather than just the pre-ART AUC viral load (Table 2). Taken together, these findings imply that replication of rebounding lineages during ATI-2 contributed to the post-ATI-2 vDNA population, but that this increase was imperceptible in the bulk CA-DNA measures.

### Replication of lineages during ATI increases likelihood of subsequent reactivation

Importantly, using this NHP model allows us to not only assess CA-vDNA measurements of reservoir but also the RCVR. We therefore tested if transient replication of rebounding variants during ATI-2 increased their probability of reactivating again during a third ATI. To this end, we discontinued treatment for a third time (ATI-3) at 682 dpi, again administering the anti-CD8α monoclonal antibody 3 days prior to ART interruption to increase viral loads during the rebound. During ATI-3, all animals rebounded within 7 days, with peak viral loads ranging from 1.2x10^3^–2.0x10^6^ copies/mL. Reactivation dynamics resembled those observed during ATI-1, with the number of barcodes detected during ATI-3 rebound viremia ranging from 12 to 38, corresponding to an estimated average reactivation rate of 4.0 (sd = 1.4) reactivation events per day (Table 1).

To investigate how viral replication prior to ATI-3 influenced reactivation, we characterized all variants found in plasma based on their cumulative peak pVL (pre-ART + ATI-1 + ATI-2), and identified which barcodes reactivated after treatment was again discontinued for ATI-3 (Fig 3 bottom panels). Consistent with previous ATIs, most lineages detected during ATI-3 were ones that were predominant in pre-ART plasma, with 58% to 83% of rebounding lineages originating from the top 10% of the pre-ART barcode distribution in each animal. The vast majority (87% to 100%) of lineages rebounding during ATI-3 had also reactivated previously during ATI-2 in each animal. However, most of these variants replicated less during ATI-2 than before the initiation of therapy, suggesting that their reactivation during ATI-3 was attributable to their large initial contribution to the RCVR in primary infection (Fig 3 bottom panels). Importantly, we also identified several variants that rebounded during ATI-3 after having replicated substantially more during ATI-2 than before therapy. Because some of these lineages were at a low frequency in pre-ART plasma, their subsequent reactivation during ATI-3 is likely attributable to replication-induced reseeding of the RCVR during ATI-2. Interestingly, all barcodes detected during ATI-3 that had not reactivated during previous ATIs were at a relatively low frequency in pre-ART plasma, raising the question of whether they had increased in frequency in the RCVR prior to ATI-3 due to other mechanisms, such as clonal expansion.

To systematically assess whether replication-induced reseeding of the RCVR occurred during ATIs, we used a model selection approach to investigate if the observed reactivation of particular lineages during ATI-3 could be explained simply by pre-ART size, or whether the replication of these lineages during earlier ATIs increased their likelihood of rebound. We treated reactivation as a binomial process with the number of trials corresponding to the number of cells harboring stable residual virus and estimated the probability of reactivation per trial via maximum likelihood for each animal. We assumed that the number of cells harboring a specific barcode is proportional to (1) the pre-ART viral load AUC attributable to that lineage (*pre-ART model*) or (2) the cumulative replication of the variant during primary infection and across the first and second ATIs (*reseeding model*). To quantitatively compare how well the two models explained the rebound data in each animal, we computed the relative likelihood of their Akaike Information Criterion (AIC) statistics. The reseeding model explained the rebound significantly better than the pre-ART model for all animals (relative likelihood > 0.95; Table 2), indicating that replication of rebounding lineages increased their probability of reactivation during subsequent ATI.

The above model analysis weighted the contribution of replicating virus to the RCVR the same way, regardless of whether replication occurred prior to ART or during an ATI. To investigate whether transient replication of virus during ATIs seeded the RCVR more or less efficiently than pre-ART replication, we also explored models where replication could be weighted differently during ATIs than prior to ART (i.e. where pVL during ATIs contributed fewer or more infected cells to the RCVR than pre-ART pVL). However, we did not find that these models improved the fit to the rebound data in any animal (Table 2).

While reactivation is presumed to be stochastic at the level of individual cells, variants that seed more cells in the RCVR should have more opportunities to reactivate close to the time of ART discontinuation and may therefore contribute more to viral rebound. Tracking the replication of individual lineages across pre-ART and subsequent ATIs revealed that barcode lineages that were predominant in pre-ART plasma were often also among the most predominant lineages during subsequent ATIs (S1 Fig). However, the contribution of individual lineages to rebound viremia was highly variable across ATIs. We further assessed if past replication of variants (pre-ART and during the previous ATI) was predictive of their level of replication during ATI-3. Consistent with previous ATIs, the relative contribution of rebounding lineages to rebound viremia during ATI-3 was significantly correlated with their relative abundance in pre-ART plasma for three of the animals (H860: r = 0.57, p = 9E-4; H814: r = 0.65, p = 9E-6; DFR6: r = 0.97; p = 9E-5; Pearson correlation; S2 Fig). However, cumulative replication of barcodes (during previous ATIs and pre-ART) was less predictive of their level of replication during ATI-3 in three of the animals than replication pre-ART alone (S1 Table). When considered independently, replication in ATI-2 did not contribute significantly to the regression model in any animal. Overall, these data present a nuanced picture of how replication during ATIs can alter the dynamics of reactivation and viral outgrowth when treatment is subsequently withdrawn. While barcode lineages that replicated substantially during ATI-2 were more likely to reactivate again during ATI-3, consistent with replication-induced reseeding of the RCVR, the outgrowth of rebounding lineages during ATI-3 was primarily influenced by their relative abundance in pre-ART plasma, presumably reflecting their initial contribution to the RCVR when it was first established in primary infection. Therefore, while the RCVR can be reseeded, the absolute levels of reseeding seem small compared to the overall reservoir size established prior to ART.

### Viral DNA reflects replication-induced reseeding of minor lineages in the RCVR

**Fig 5:**
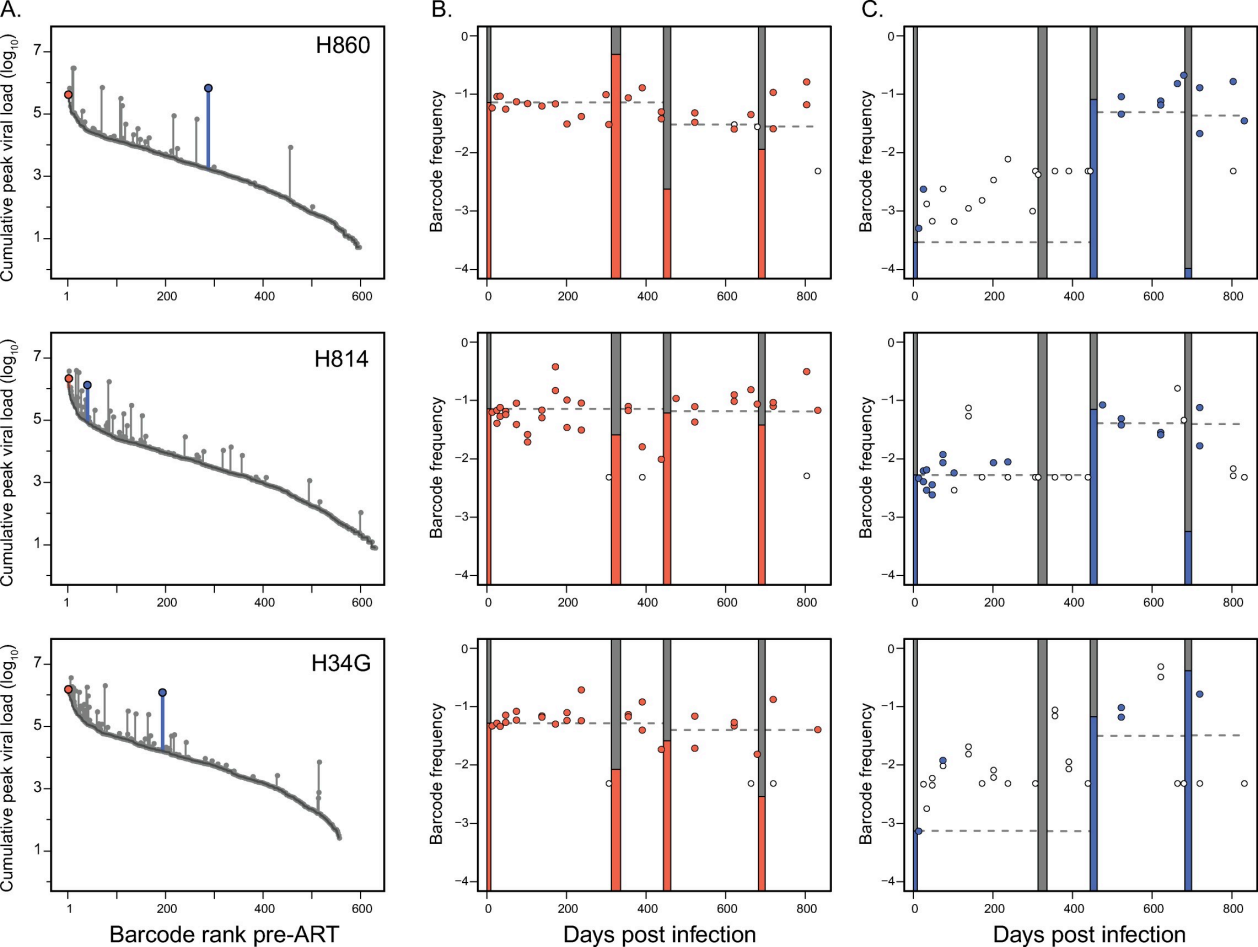
Frequency of rebounding lineages in CA-vDNA on ART reflects replication during ATIs. The relative DNA frequencies of select lineages that rebounded in ATI-3 are tracked in vDNA on ART for animals H860, H814, and H34G. In panel (A), the grey points correspond to the cumulative peak viral loads of barcodes, rank-ordered based on their relative frequency at d10. The dark grey lines depict the pre-ART barcode distribution, with increases in total viral load highlighted by vertical line segments. Select lineages that were dominant in pre-ART plasma viremia but subsequently increased negligibly in total viral load are highlighted in red, while variants with over 10-fold higher level of replication during ATI-2 than primary infection are highlighted in blue. In panel (B), the red filled circles depict the relative frequencies of predominant barcodes in vDNA while the open circles indicate the limit of detection at time points when a particular barcode was not observed. The grey bars highlight the time intervals when the animals were off therapy, with the red bars indicating the relative frequency of the barcode at peak viremia during each interval. In panel (C), the blue filled circles depict the vDNA frequencies of the lineages that replicated substantially during ATI-2, while the open circles indicate the limit of detection at time points when a particular barcode was not observed. The grey bars again highlight the time intervals when the animals were off therapy, with the blue bars indicating the relative frequency of that barcode at peak viremia during each interval. The dashed lines indicate the relative frequency of each lineage based on cumulative peak plasma viral load.

Since persistent viral DNA is the most readily measurable surrogate marker for the RCVR in HIV infected individuals, we further assessed whether the replication of rebounding variants during ATI-2 was reflected in the viral DNA population (Fig 5). We identified several rebounding lineages that were sampled in PBMC DNA on ART at multiple time points throughout the study and tracked how their relative frequency changed in response to transient replication during successive ATIs. Overall, the contribution of variants to CA-vDNA mirrored their replication in plasma before ART and during ATIs. Prior to ATI-2, the frequency of lineages in CA-vDNA closely matched their frequency in pre-ART plasma, indicating that replication of each lineage before initiation of ART determined their initial contribution to viral DNA. Several variants that did not replicate substantially during ATI-2 remained at an approximately constant DNA frequency throughout the study, consistent with their overall level of viral replication (Fig 5B). By contrast, lineages that replicated over 10-fold more during ATI-2 than before ART exhibited commensurate, persistent, increases in vDNA frequency (Fig 5C). Importantly, subsequent reactivation of these variants after treatment was again discontinued definitively demonstrated that transient replication during ATI-2 reseeded the RCVR.

**Fig 6:**
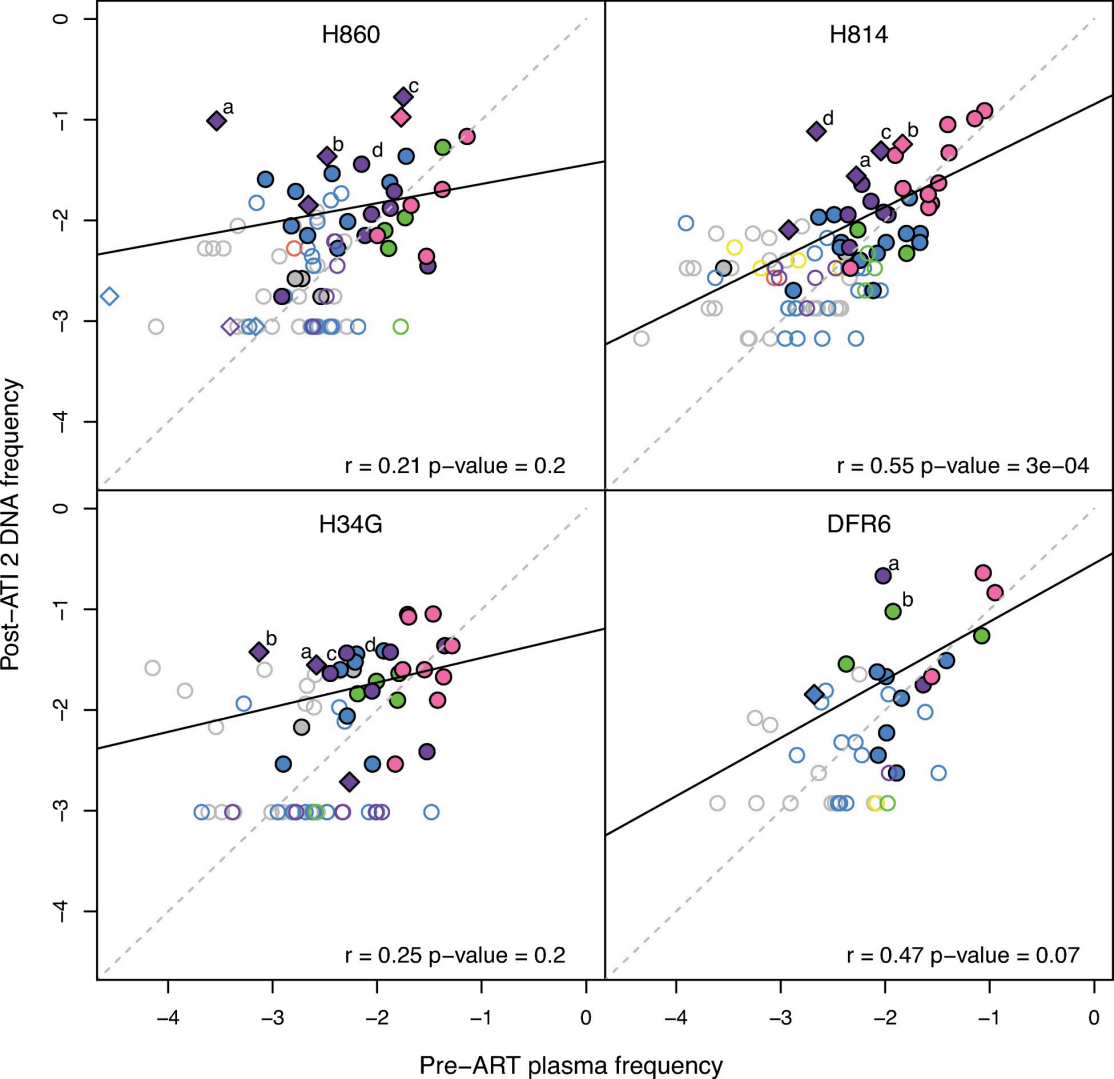
Changes to the composition of the PBMC CA-vDNA population. The estimated average DNA frequencies of all barcodes in post-ATI-2 on-ART samples compared to their relative frequencies in pre-ART plasma. The open symbols designate barcodes detected only at a single time point. The diamonds highlight barcodes that replicated at least 10-fold more in ATI-2 than pre-ART. The color coding designates which ATIs the barcodes were detected in: no ATI (grey), ATI-1 only (yellow), ATI-2 only (blue), ATI-3 only (red), ATI-1 and ATI-2 (green), ATI-2 and ATI-3 (purple), all ATIs (pink). The dark solid lines depict the linear regressions, while the grey dashed lines represent theoretical one-to-one correspondence.

To better understand how the composition of the proviral DNA population changed overall during the course of infection, we compared the average PBMC vDNA frequencies of all barcode lineages sampled on-ART after ATI-2 to their relative abundance in pre-ART plasma viremia (Fig 6). Individual lineage frequencies were not significantly correlated between pre-ART plasma and on-ART PBMC vDNA, apart from a single animal (H814: r = 0.55; p = 0.0003). Importantly, we observed several lineages that increased markedly in relative PBMC vDNA frequency after ATI-2 relative to their pre-ART plasma levels. As shown above for selected examples (Fig 5C), the increase in PBMC vDNA frequency was commensurate with the level of replication during ATI-2 for many of these variants, indicating that transient replication altered their contribution to the CA-vDNA population (Figs 6 and S3–S5). However, several lineages exhibited relative frequency profiles consistent with clonal expansion, increasing substantially in PBMC vDNA frequency despite replicating marginally, or not reactivating at all during ATI-2 (Figs 6, S3, S4, and S6). Furthermore, two barcode lineages that constituted minor lineages in pre-therapy plasma increased in frequency prior to reactivating in ATI-2, after which they persisted in PBMC vDNA at a level consistent with their overall replication (Figs 6, S4 and S6). In general, however, limited sampling of individual barcode lineages across multiple time points precluded deconvoluting the putative effects of replication-induced reseeding and clonal expansion on their dynamics in the viral DNA population. Overall, we found that viral replication during an ATI can alter the genetic constituents of the viral DNA population despite not increasing its absolute size as assessed by total SIV DNA measurements. Although these differences might not be readily detectable in HIV infected individuals, it is important to understand this possibility when initiating ATIs in humans, particularly if the ATI will occur for an extended period and/or allow substantial viral replication while awaiting equilibration to a post-ART “setpoint” (post-treatment control).

## Discussion

Clinical trials currently require the use of ATIs to assess whether proposed interventions to target persistent virus can increase time to rebound or achieve control of viremia without ART. However, concerns remain about whether rebounding lineages that grow out during ATIs can reseed the RCVR, potentially impacting the efficacy of future treatment strategies, particularly for people who started ART early after infection and are likely to have small RCVRs [43,44]. While the effects of short-term ATIs on the rebound-competent viral reservoir appear to be limited based on assessment of total, intact and replication-competent vDNA populations [16–19], due to sampling and assay limitations it is challenging to assess the size and genetic composition of the RCVR based on these indirect surrogates [8,25,45]. To directly determine if transient rebound viremia can reseed the RCVR, we used a barcoded virus in an NHP model to quantify the replication of individual viral lineages before initiation of ART and through a series of three successive treatment interruptions. This new approach allowed us to demonstrate that replication of rebounding lineages during viral recrudescence increased their likelihood of reactivating again during a subsequent ATI. We observed enrichment of the RCVR with rebounding lineages when rebound viremia was comparable to that of primary infection, but not when viral replication was more limited. Our findings corroborate previous studies demonstrating that ATIs that limit total viral replication by brief duration and/or re-imposition of ART after limited measured plasma viremia, do not meaningfully impact the RCVR [18], but suggest that reseeding of the RCVR if replication is more robust can alter its genetic composition, which may have important implications for future, long-term treatment interruption studies. While our model utilized transient experimental depletion of CD8α+ cells to boost viral replication in a short time period, long-term off-ART protocols, which can generate high total viral replication levels, might also alter the composition of the RCVR.

Tracking individual viral lineages across a series of treatment interruptions revealed that rebounding lineages were seeded in the RCVR proportionately to their level of replication during the ATI. Consequently, the potential for expansion of the RCVR after discontinuation of treatment depends directly on the level of rebound viremia reached before re-suppression. If viral replication during an ATI is limited, the additional contribution of rebounding lineages to the RCVR should be negligible, explaining why reactivation of barcode lineages during ATI-1 was not predictive of their subsequent detection in DNA after treatment was continued. However, even if the overall level of transient replication reached during an ATI is high, individual rebounding lineages can only become substantially enriched in the RCVR if they replicate more during the ATI than when the RCVR was first established. Because most variants that reactivated during ATI-2 were already predominant pre-ART, their potential for reseeding the RCVR was eclipsed by their initial contribution to the RCVR before therapy. Consequently, we did not observe increases in their PBMC vDNA frequencies. However, we also identified several lineages that replicated 10 to 100-fold more during ATI-2 that before ART, presumably due to having reactivated closer to the time of ART discontinuation and outgrown than other lineages that reactivated later during the same ATI. Importantly, many of these lineages also increased substantially in CA-vDNA frequency after ATI-2 and reactivated again during ATI-3, demonstrating that they were enriched in the RCVR due to replication-induced reseeding during ATI-2.

While replication of individual viral lineages before ART initiation determined the genetic composition of the RCVR when it was first established, cellular proliferation of resting CD4+ cells harboring replication-competent viral genomes is thought to constitute an important mechanism driving the persistence of the RCVR during long-term ART in both HIV-infected humans and SIV-infected macaques [45–50]. Although we could not directly assess the contribution of clonal expansion to the RCVR due to the overall low level of the reservoir, tracking PBMC vDNA frequencies of individual barcode lineages longitudinally revealed patterns consistent with clonal expansion. We observed several barcode lineages that increased markedly in PBMC vDNA frequency despite not replicating substantially, or reactivating at all, during treatment interruptions. Furthermore, several viral variants that were at a relatively low frequency in pre-ART plasma nonetheless reactivated for the first-time during ATI-3. However, we did not detect these lineages in the viral DNA population and could not determine if they had increased in frequency prior to ATI-3. Limited sampling depth restricted our analysis to the most predominant barcode lineages in the proviral DNA population, most of which remained at a constant frequency in PBMC vDNA, consistent with their cumulative level of replication pre-ART and during ATIs. While future studies utilizing integration site analysis are required to resolve the contribution of clonal expansion to the RCVR during ART, our study showed that viral replication, before ART and during ATIs, regardless of its source, plays a central role in determining the genetic structure of the RCVR.

Our demonstration that the RCVR is seeded with rebounding lineages during viral recrudescence proportionately to their level of replication implies that replication during ATIs can increase the absolute size of the RCVR. However, despite the high viral loads reached during ATI-2, overall viral DNA levels increased only transiently and returned rapidly to pre-ATI levels. A lack of increase in CA-vDNA levels in previous studies of HIV-infected individuals has been interpreted as evidence that viral replication during short-term ATIs does not substantially expand the RCVR [13,16,18,51]. However, the persisting HIV-1 vDNA population is dominated by defective proviruses that are not relevant for viral rebound [8,23–25], which makes interpretation of this measure as a surrogate for RCVR size challenging. In contrast to HIV-1, in the model employed here the majority of persistent vDNA remains intact in SIV-infected macaques after 18 months of ART and may therefore reflect the RCVR better than in HIV-1 infected individuals [52,53]. A recent study utilizing the intact proviral DNA assay [54], which provides a more relevant measure for the size of the RCVR in HIV-1 infected individuals [55], found that people who experienced frequent, non-structured treatment interruptions were significantly more likely to have larger intact proviral DNA reservoirs on-ART [56]. These results suggest that substantial replication off-ART may be required to detect differences in the size of the RCVR. In our study, total viral replication across ATIs was equivalent to that in primary infection, suggesting that replication-induced reseeding of the RCVR during ATIs could have at most doubled its size. Taken together, these observations suggest that detecting nuanced changes to the size of the persisting SIV DNA population may not be feasible through bulk CA-vDNA measures, however, the increased analytic resolution provided by tracking individual barcoded lineages in PBMC vDNA provides a way to indirectly assess changes to its overall size.

Several studies have observed an intriguing lack of concordance between virus variants that emerged after treatment discontinuation and sequences obtained from pre-ATI QVOA and intact proviral DNA populations [18,28–31]. In the present study, replication of viral variants in primary infection was a strikingly strong predictor of their subsequent reactivation after treatment discontinuation, with rebound primarily involving lineages that were predominant in pre-ART plasma. However, despite the increased analytic resolution afforded by deep sequencing of the short genetic barcode in the SIVmac239M genome, most lineages rebounding during ATI-2 were not detected in on-ART PBMC vDNA prior to the ATI, consistent with previous findings in HIV-1 infected individuals [18,28–31]. Importantly, the frequency of barcode lineages sampled in on-ART PBMC vDNA was correlated with their relative abundance in pre-ART plasma, indicating that the composition of the proviral DNA population mirrored that of the virus population replicating before therapy. Therefore, more frequent sampling would likely have uncovered additional minor barcode lineages in pre-ATI PBMC vDNA that subsequently reactivated after treatment discontinuation [35]. Overall, our findings highlight the challenges posed by limited sampling depth for characterizing the composition of the vDNA population on ART and predicting which of these lineages rebound after treatment discontinuation. In addition to sampling limitations, potential selection during rebound for rare viral phenotypes in the RCVR could further contribute to the observed discrepancy between rebounding lineages and those sampled from QVOA and proviral DNA populations in HIV-1 infected individuals [57]. The present study was designed to minimize phenotypic differences between virus variants that could confound rebound dynamics by using a viral stock composed of virus variants which were isogenic outside of the barcode sequence and initiating ART shortly after infection to minimize accumulation of mutations in the RCVR. However, the diversified viral swarms typical of HIV infected individuals have significant viral phenotypic differences, which may lead to differential outgrowth dynamics between reactivating variants.

Because barcoded variants have similar replicative capacity, we expect that their individual contributions to rebound viremia depend primarily on when they reactivated relative to ART discontinuation (i.e. their duration of exponential growth). Interestingly, the contribution of reactivating lineages to rebound viremia during each ATI was primarily determined by their relative abundance in pre-ART plasma (apart from ATI-3 for animal H34G), rather than their total replication during previous ATIs and pre-ART. This implies that virus variants that predominated in early infection were among the first to reactivate after treatment discontinuation. One possible explanation for this phenomenon is that the RCVR may include a limited and saturable subset of highly susceptible and reactivable cells that gets rapidly filled up by those lineages that replicate the most during the earliest days of infection [36]. In this scenario, while infection of less reactivable cells would continue to expand the RCVR and contribute to the persisting DNA population during primary infection and ATIs, rebound would primarily initiate from the smaller subset of cells more likely to reactivate shortly after treatment discontinuation. A heterogeneous RCVR could explain why replication of rebounding lineages during ATI-2 increased their likelihood of reactivating again during ATI-3 but not their contribution to rebound viremia, which was primarily determined by replication pre-ART. While speculative, this hypothesis is supported by our previous discovery that the recrudescence rate saturates within days of infection, even though continued viral replication substantially increases levels of persistent SIV DNA. However, a specific cellular or tissue compartment that could serve as the primary source of rebound virus has not been identified [32,33]. Characterizing the anatomical distribution and heterogeneity of the tissue milieu in which the RCVR resides is critical to resolving the dynamics underlying its formation, maintenance, and reactivation.

In the present study, we depleted animals of CD8α+ cells prior to the second and third ATIs to increase viral replication so that potential enrichment of rebounding lineages in the RCVR would be detectable. Our motivation for this experimental approach was to achieve high levels of viral replication in a short period of time to prevent the accumulation of fitness differences between variants that could confound analysis of rebound dynamics. However, in addition to increasing viral replication, CD8 depletion may have induced broader reactivation of cells in the RCVR due to CD4 proliferation [41], which has important implications for the translatability of our findings to ATIs in people living with HIV. In particular, the reactivation of numerous minor barcode variants during ATI-2, which allowed us to detect reseeding of the RCVR, was likely generated by the experimentally mediated high reactivation rates due to CD8 depletion. Furthermore, if replication-induced reseeding of rebounding barcode lineages during ATI-2 contributed less reactivable cells to the RCVR than those seeded during the earliest days of infection, it is possible that their subsequent reactivation during ATI-3 would not have occurred without prior CD8 depletion. Lower reactivation rates in people likely lead to substantially less frequent reactivation of minor RCVR variants even during long-term ATIs [58]. Even if rare pre-existing viral phenotypes became enriched in the RCVR after rebounding, they would likely not impact the efficacy of future cure intervention strategies unless they already harbored specific mutations that would interfere with these therapies. However, the emergence and selection of immune escape mutations during long-term ATIs in people may lead to enrichment in the RCVR. Even if replication-induced reseeding during ATI involves less reactivable cells than those that established the RCVR pre-ART, the former may initiate viral rebound if immune selection prevents the outgrowth of wildtype virus seeded earlier in infection. The potential for enrichment of the RCVR with escape mutations emerging during long-term ATIs may therefore hinder future therapies aimed at harnessing host immune responses to induce a functional cure.

Overall, our study highlights the increased power and sensitivity of tracking individual viral lineages rather than the virus population as a whole to investigate the dynamics underlying the formation of the RCVR during primary infection, its maintenance during suppressive ART, and its reactivation and replenishment after treatment discontinuation. Our discovery that viral replication during treatment interruptions can alter the RCVR has important implications for individuals undergoing long-term ATIs and underscores the importance of NHP studies for assessing the safety of clinical trials.

## Methods

### Ethics statement

4 purpose-bred Indian-origin male rhesus macaques (*Macaca mulatta*) were housed at the National Institutes of Health (NIH) in a facility that is accredited by AAALAC International and follows the Public Service Policy for the Care and Use of Laboratory Animals. The Institutional Animal Care and Use Committee for the National Cancer Institute (NCI, USA) approved the use of nonhuman primates for this research. Animal care was provided in accordance with the procedures outlined in “The Guide for the Care and Use of Laboratory Animals”. At the start of the study, all animals were free of cercopithecine herpesvirus 1, simian immunodeficiency virus (SIV), simian type-D retrovirus, and simian T-lymphotropic virus type 1. All animals were treated with enrofloxacin (10 mg/kg once daily for 10 days), paromomycin (25 mg/kg twice daily for 10 days), and fenbendazole (50 mg/kg once daily for 5 days) followed by weekly fecal culture and parasite exams for 3 weeks to ensure they were free of common enteric pathogens. At least a 4-week post-treatment period allowed time for stabilization of the microbiome prior to use in this study.

### Animal study

Animals were each intravenously inoculated with 1x10^4^ infectious units of transfection produced SIVmac239M [26]. At 10 days post-infection, the animals were started on a combination antiretroviral therapy (ART) regimen consisting of a co-formulated triple regimen containing tenofovir disoproxil fumarate (TDF; 5.1 mg/kg)(Hangzhou APIChem Technology), Emtricitabine (FTC; 40 mg/kg)(Hangzhou APIChem Technology) and dolutegravir (DTG; 2.5 mg/kg)(kindly provided by ViiV Healthcare) administered by once-daily subcutaneous injection. The drug regimen was formulated and administered as previously described [59]. A series of 3 short-term treatment interruptions were performed, with therapy first discontinued at 313 dpi in all animals and resumed at 332 dpi in animal DFR6 and at 336 dpi in the remaining three animals. Therapy was next withdrawn at 444 dpi and continued at 462 dpi in all animals, followed by a final treatment interruption between 682 dpi and 699 dpi, with the animals remaining on ART until 843 dpi. The last two treatment interruptions were preceded at 441 dpi and 679 dpi by administration of a single dose of the rhesusized anti-CD8α antibody, MT-807R1 (NIH Nonhuman Primate Reagent Resource), at 50 mg/kg SubQ.

### Sample collection and processing

Whole blood was collected from sedated animals. Plasma for viral RNA quantification and sequencing and PBMCs for flow cytometric assays and cell-associated viral DNA quantification were prepared from blood collected in EDTA Vacutainer tubes (BD). Following separation from whole blood by centrifugation, plasma aliquots were stored at -80°C. After plasma separation, the cellular fraction of each whole blood sample was resuspended in PBS and PBMCs were then isolated by Ficoll-Paque Plus (GE Healthcare) gradient centrifugation.

### Plasma viral loads

Quantitative real-time PCR was used to quantify SIV RNA plasma viral loads (with a limit of quantification of 15 viral RNA copies per milliliter) at all sampled time points as described previously [60].

### Quantitative evaluation of cell-associated DNA and RNA

Quantitative assessment of cell-associated viral DNA in PBMC pellets was determined by the hybrid real-time/digital RT-PCR and PCR assays essentially as described in [61,62] but specifically modified to accommodate cell pellets as described in [26]. Limit of detection was evaluated on a sample by sample basis, dependent on the number of diploid genome equivalents of extracted DNA assayed.

### Miseq sample preparation

RNA samples were prepared for MiSeq sequencing as previously described [26]. RNA isolation from plasma was performed using a QIAamp Viral RNA mini kit. Superscript III reverse transcriptase (Invitrogen) and a reverse primer (Vpr.cDNA3: 5’-CAG GTT GGC CGA TTC TGG AGT GGA TGC-3’ at position 6406–6380) were used to synthesize cDNA. The cDNA was quantified via qRT-PCR using the primers VpxF1 5’-CTA GGG GAA GGA CAT GGG GCA GG-3’ at 6082–6101 and VprR1 5’-CCA GAA CCT CCA CTA CCC ATT CATC-3’ at 6220–6199. The isolation and quantification of viral DNA from PBMC samples was performed as previously described [26]. PCR prior to sequencing was performed with VpxF1 and vprR1 primers combined with either the F5 or F7 Illumina adaptors containing unique 8-nucleotide index sequences for multiplexing. PCR was performed using High Fidelity Platinum Taq (ThermoFisher) under the following conditions: 94°C, 2 min; 40 × (94°C, 15 s; 60°C, 1.5 min; 68°C, 30 s); 68°C, 5 min. The multiplexed samples and *Phi X 174* library were prepared via standard protocols and sequenced on a MiSeq instrument (Illumina). The number of input templates for plasma RNA samples ranged from 274 copies to 1.6 × 10^6^ copies. For CA-DNA from PBMCs, the number of quantified input templates ranged from 2.3 to 3.6x10^4^ copies but was not quantifiable for each sample.

### Single genome amplification

For low-template samples, we used single genome amplification (SGA) followed by direct Sanger sequencing to assess the frequency and number of unique barcodes. cDNA synthesis and PCR were performed as described above but using a limiting dilution of cDNA or DNA prior to PCR amplification.

### Sequencing analysis

Sequences were demultiplexed into individual samples based on exact matches to the Illumina P5 index. After barcode splitting, sequences were aligned to the first 28 bases of the vpr gene allowing for 2 nucleotide mismatches. The 34 bases directly upstream of the start codon for vpr were extracted, corresponding to the barcode. Since the number of template cDNA copies was quantified using qRT-PCR, the theoretical limit of detection was estimated for each sample as the minimum number of sequences that would result from a single copy of an input template. Sequences below this threshold were discarded. Only identical matches to a defined barcode in the SIVmac239M stock were counted as an authentic input sequences, apart from single unique sequence in each of animals H860 and H814 that were predominant (>0.1% frequency) in the pre-ART samples. In rare cases, all minor barcode sequences that differed at a single nucleotide from a more prevalent barcode (at least 100-fold higher in frequency) in the same sample were excluded. Index hopping was observed infrequently between multiplexed samples, with index-hopped sequences excluded. To facilitate tracking individual viral lineages across the entire study, 2 barcode lineages in each of animals H814 and H34G that were above the limit of detection during ATI-2 but not observed in pre-ART plasma were excluded from analysis.

### Statistical analyses

All statistical analysis and computational simulations were performed in R version 3.6.3. The maxLik package was used for model fitting and the DescTools package was used for calculating AUC of viral loads.

### Estimation of reactivation rates

We estimated the reactivation rate during each ATI using a previously described method that incorporates the relative abundance of the rebounding variants and the growth rate of the virus [26,37]. Assuming that each barcode lineage grows exponentially at approximately the same rate, the reactivation rate can be estimated as

$$RR=\frac{g(n-1)}{\sum_{i=1}^{n-1}\ln{(S}_{i+1}-S_{i})},$$

where n is the number of barcodes, *g* is the growth rate, and ***S_i_*** is the number of sequencing reads of barcode *i* at peak rebound viremia. For each ATI, we estimated the growth rate as the average of the maximal two-point growth rate estimates of each animal. For animal DFR6, we observed a biphasic viral load profile during ATI-3 with several distinct lineages detected at each peak (at 686 dpi and at 693 dpi). We estimated the reactivation rate based on the higher viral load peak at 693 dpi.

### Estimating mean variant frequency in PBMC vDNA

A binomial sampling model was used to estimate the average frequency of a variant across a set of PBMC samples. The probability that a particular barcode at relative frequency p in the PBMC CA-DNA population is observed at proportion q in a given sample was assumed to follow a binomial distribution, with the number of trials corresponding to the number of input templates S in the sample and the number of successes k to the maximum integer value of qS. The log-likelihood of the observed frequencies of a given barcode in n samples is defined as l(k,S)=∑i=0nlog(Siki)+kilogp+(Si−ki)log(1−p). A maximum likelihood approach based on the BFGS algorithm (implemented in the maxLik package in R) was used to estimate the probability of success per trial p, i.e. the average frequency of the barcode in PBMC vDNA. For samples in which the number of input templates was undetectable using QT-PCR, the limit of detection was set to 200 copies/mL. Both negative (barcode not detected) and positive (barcode detected at some frequency) samples were used in the calculations, however, the method was used only for variants that were detected in at least one PBMC samples.

### Calculation of area under curve of viral load

The total area under the viral load curve was estimated for off-ART cycles (pre-ART, ATI-1 and ATI-2) using the trapezoidal method as implemented by the AUC function of the DescTools R package. For ATIs, we included the last sampled time point with undetectable viral load in the calculations, assuming the viral load was 14 SIV RNA copies/mL.

### Reactivation of variants based on replication pre-ART

To assess how the probability of reactivation of barcoded variants during ATI-2 was related to their level of replication at time of ART initiation, the pre-ART barcode peak viral loads were partitioned into 0.25-log_10_ intervals, with the proportion of barcodes that rebounded during ATI-2 computed for each viral load category. Computational simulations were then used to determine the proportion of lineages expected to reactivate from each pre-ART viral load category for each animal, under the assumption that replication before ART was directly proportional to the probability of reactivation. During each simulation, the set of lineages detected in pre-ART plasma was sampled without replacement until the number of sampled barcodes matched the number of reactivated lineages detected during ATI-2 for each animal, with the probability of sampling a given barcode determined by its relative frequency pre-ART. 1000 simulations were performed for each animal, and the median and 90% credibility intervals for the proportion of variants reactivating from each viral load category were computed. The Chi-Square goodness-of-fit test was used to assess if the observed proportions of variants reactivating from each viral load category were significantly different from the simulated median proportions of reactivating lineages (chisq.test in R, with simulated p-value based on 2000 replicates).

### Detection of barcode variants in CA-vDNA based on viral replication

Logistic regression was used to assess if rebounding lineages were enriched in PBMC CA-vDNA after ATI-1 and ATI-2. For ATI-1, we assessed if accounting for whether variants reactivated during ATI 1 better explained their detection in at least one post-ATI-1 PBMC sample (346–443 dpi) than their pre-ART peak viral loads alone. The log-odds of detecting a barcode based on the values of the explanatory variables for the baseline and alternative models are defined as ***l*_1,*B*_ = *β*_0_+*β*_1_*x*_1_** and ***l*_1,*R*_ = *β*_0_+*β*_1_*x*_1_+*β*_2_*x*_2_**, respectively, where ***x*_1_** is the pre-ART lineage peak viral load (log10) and ***x*_2_** is a binary variable indicating whether the lineage reactivated during ATI-1. For ATI-2, we assessed if the cumulative total viral replication of barcodes pre-ART and during ATI-1 and ATI-2 better explained their detection in at least one post-ATI-2 PBMC sample (552–682 dpi) than their total replication pre-ART alone. The log-odds of detecting a barcode based on the values of the explanatory variables for the baseline and alternative models for ATI-2 are defined as ***l*_2,*B*_ = *β*_0_+*β*_1_*x*_1_** and ***l*_2,*R*_ = *β*_0_+*β*_3_*x*_3_**, respectively, where ***x*_1_** is the pre-ART barcode AUC of total viral load multiplied by the relative frequency of the variant at peak viremia and ***x*_3_** is the sum of pre-ART, ATI-1 and ATI-2 lineage AUC of viral load. To assess which model better explained the detection of variants after each ATI, the relative likelihood e^(AICmin-AICmax)/2^ of the two models was computed based on their AIC scores.

### Modeling reactivation as a function of viral replication

A model selection approach was used to assess if viral replication during treatment interruptions contributed significantly to the RCVR or conversely, if the composition of the RCVR was set in primary infection prior to initiation of ART and remained unaltered thereafter. Reactivation during the final ATI was modeled as a binomial process with the number of trials proportional to the number of cells harboring virus in the RCVR and a constant probability of reactivation per cell. In the *pre-ART model*, the number of trials **N_i_** for each lineage was set to the maximum integer value of the viral load attributable to that lineage in pre-ART plasma viremia (AUC of total viral load multiplied by the relative frequency of the barcode at peak viremia). In the *reseeding model*, the number of trials for each variant was set to the maximum integer value of the cumulative viral load attributable to the variant, **V_0,i_+V_1,i_+V_2,i_**, where **V_0,i_**, **V_1,i_** and **V_2,i_** correspond to the barcode proportion at peak viremia multiplied by AUC of viral load before therapy, during ATI-1, and during ATI-2. In the *weighted reseeding model*, the relative contribution of viral load during ATI-1 and ATI-2 to the RCVR was increased β - fold, with the number of trials for each lineage set to the maximum integer value of **V_0,i_**+β(**V_1,i_+V_2,i_**). The log-likelihood of the data for each model is defined as $LL=\sum_{i=1}^{R}log(1-{\left(1-p\right)}^{N_{i}})+\sum_{i=1}^{U}N_{i}log(1-p),$ where p is the probability of reactivation per trial, R is the number of lineages rebounding during ATI-3 and U is the number of barcodes observed in pre-ART plasma but not during ATI-3 rebound. A maximum likelihood approach based on the BFGS algorithm (implemented in the maxLik package in R) was used to estimate the parameters of each model. To assess which model best explained the rebound data in each animal, i.e. which pre-therapy lineages reactivated and did not reactivate during ATI 3, the relative likelihood e^(AICmin-AICmax)/2^ of each pair of models was computed based on their AIC scores.

### Replication of barcodes in ATI-3 based on past viral replication

Linear regression was used to assess if the viral load attributable to individual rebounding lineages during ATI-3 peak rebound viremia was predicted by their past level of replication. We considered the following three different models: (1) replication of barcodes pre-ART (AUC of viral load multiplied by the relative frequency of the barcode at peak viremia) as the sole explanatory variable; (2) cumulative replication of barcodes prior to ATI-3 (sum of pre-ART, ATI-1 and ATI-2 barcode AUC of viral load) as the sole explanatory variable; (3) replication of barcodes pre-ART (AUC of barcode viral load) and replication of barcodes in ATI-2 (1 + AUC of barcode viral load) as separate covariates. Analysis was restricted to those barcodes that rebounded during ATI-3 and viral load AUC values were log10-transformed, with ATI-2 AUC viral loads log10(x+1)-transformed to prevent undefined values for barcodes that did not reactivate in ATI-2. We compared the models based on their R-squared values and the statistical significance of the estimated regression coefficients. For animal DFR6, only barcodes detected at peak rebound viremia during ATI-3 were included in the analysis.

## Supporting information

S1 Table Predictors of barcode replication during ATI-3. Linear regression models for replication of rebounding lineages during ATI-3 based on past replication pre-ART and during ATIs. The sole explanatory variable for the pre-ART model is the pre-ART barcode-specific AUC of virus; the total virus replication model has the sum of barcode-specific AUCs of virus (during pre-ART, ATI-1 and ATI-2) as the explanatory variable. The pre-ART and ATI-2 model includes pre-ART barcode AUC of virus and ATI-2 barcode AUC of virus as independent covariates to allow differential effects of replication during these periods.
(TIF)

S1 Fig Replication of lineages pre-ART and across ATIs. Grey circles indicate viral load (log_10_) attributable to all barcodes detected in pre-ART plasma. Variants that reactivated during any subsequent ATI are outlined in black. Yellow circles correspond to individual barcodes that were ≥ 1% frequency both pre-ART and during ATI-2, blue circles indicate barcodes ≥ 1% in pre-ART plasma only, red circles indicate barcodes ≥ 1% during ATI-2 only, and green circles indicate barcodes that did not reactivate prior to ATI-3. Cyan lines connect barcodes that were detected across all ATIs while green lines connect barcodes that were only detected in ATI-3.
(TIF)

S2 Fig Correlation of lineage-specific viral load between plasma pre-ART and each ATI. The viral load (log_10_) attributable to each lineage detected both in pre-ART plasma and during each ATI are shown in blue, while variants not detected during the ATI are shown in grey. The dashed lines depict the linear regression fits.
(TIF)

S3 Fig Barcode PBMC vDNA profiles consistent with reseeding or clonal expansion for animal H860. The colored filled circles depict the relative frequencies of the barcodes in viral DNA while the grey circles indicate the limit of detection at time points when a particular variant was not observed. Open circles correspond to the maximum limit of detection for samples where input was not quantifiable. The grey bars highlight the time intervals when the animals were off therapy, with the colored bars indicating the relative frequency of the variant at peak viremia during each interval. The grey dashed lines indicate the relative frequency of each barcode based on cumulative peak plasma viral load. The dashed blue lines indicate the average barcode frequency in PBMC across all post-ATI-2 samples, estimated via maximum likelihood.
(TIF)

S4 Fig Barcode PBMC vDNA profiles consistent with reseeding or clonal expansion for animal H814. The colored filled circles depict the relative frequencies of the barcodes in vDNA while the grey circles indicate the limit of detection at time points when a particular variant was not observed. Open circles correspond to the maximum limit of detection for samples where input was not quantifiable. The grey bars highlight the time intervals when the animals were off therapy, with the colored bars indicating the relative frequency of the variant at peak viremia during each interval. The grey dashed lines indicate the relative frequency of each barcode based on cumulative peak plasma viral load. The dashed blue lines indicate the average barcode frequency in PBMC across all post-ATI-2 samples, estimated via maximum likelihood.
(TIF)

S5 Fig Barcode PBMC vDNA profiles consistent with reseeding or clonal expansion for animal H34G. The colored filled circles depict the relative frequencies of the barcodes in vDNA while the grey circles indicate the limit of detection at time points when a particular variant was not observed. Open circles correspond to the maximum limit of detection for samples where input was not quantifiable. The grey bars highlight the time intervals when the animals were off therapy, with the colored bars indicating the relative frequency of the variant at peak viremia during each interval. The grey dashed lines indicate the relative frequency of each barcode based on cumulative peak plasma viral load. The dashed blue lines indicate the average barcode frequency in PBMC across all post-ATI-2 samples, estimated via maximum likelihood.
(TIF)

S6 Fig Barcode PBMC vDNA profiles consistent with reseeding or clonal expansion for animal DFR6. The colored filled circles depict the relative frequencies of the barcodes in vDNA while the grey circles indicate the limit of detection at time points when a particular variant was not observed. Open circles correspond to the maximum limit of detection for samples where input was not quantifiable. The grey bars highlight the time intervals when the animals were off therapy, with the colored bars indicating the relative frequency of the variant at peak viremia during each interval. The grey dashed lines indicate the relative frequency of each barcode based on cumulative peak plasma viral load. The dashed blue lines indicate the average barcode frequency in PBMC across all post-ATI-2 samples, estimated via maximum likelihood.
(TIF)

## References

[1] Finzi D, Hermankova M, Pierson T, Carruth LM, Buck C, Chaisson RE, et al. Identification of a reservoir for HIV-1 in patients on highly active antiretroviral therapy. Science. 1997;278(5341):1295–300. Epub 1997/11/21. doi: 10.1126/science.278.5341.1295 . 9360927

[2] Chun TW, Stuyver L, Mizell SB, Ehler LA, Mican JA, Baseler M, et al. Presence of an inducible HIV-1 latent reservoir during highly active antiretroviral therapy. Proc Natl Acad Sci U S A. 1997;94(24):13193–7. Epub 1997/12/16. doi: 10.1073/pnas.94.24.13193 ; PubMed Central PMCID: PMC24285. 9371822 PMC24285

[3] Chun TW, Davey RT Jr., Engel D, Lane HC, Fauci AS. Re-emergence of HIV after stopping therapy. Nature. 1999;401(6756):874–5. Epub 1999/11/30. doi: 10.1038/44755 . 10553903

[4] Davey RT Jr., Bhat N, Yoder C, Chun TW, Metcalf JA, Dewar R, et al. HIV-1 and T cell dynamics after interruption of highly active antiretroviral therapy (HAART) in patients with a history of sustained viral suppression. Proc Natl Acad Sci U S A. 1999;96(26):15109–14. Epub 1999/12/28. doi: 10.1073/pnas.96.26.15109 ; PubMed Central PMCID: PMC24781. 10611346 PMC24781

[5] Finzi D, Blankson J, Siliciano JD, Margolick JB, Chadwick K, Pierson T, et al. Latent infection of CD4+ T cells provides a mechanism for lifelong persistence of HIV-1, even in patients on effective combination therapy. Nat Med. 1999;5(5):512–7. Epub 1999/05/06. doi: 10.1038/8394 . 10229227

[6] Chun TW, Carruth L, Finzi D, Shen X, DiGiuseppe JA, Taylor H, et al. Quantification of latent tissue reservoirs and total body viral load in HIV-1 infection. Nature. 1997;387(6629):183–8. Epub 1997/05/08. doi: 10.1038/387183a0 . 9144289

[7] Chun TW, Engel D, Berrey MM, Shea T, Corey L, Fauci AS. Early establishment of a pool of latently infected, resting CD4(+) T cells during primary HIV-1 infection. Proc Natl Acad Sci U S A. 1998;95(15):8869–73. Epub 1998/07/22. doi: 10.1073/pnas.95.15.8869 ; PubMed Central PMCID: PMC21169. 9671771 PMC21169

[8] Ho YC, Shan L, Hosmane NN, Wang J, Laskey SB, Rosenbloom DI, et al. Replication-competent noninduced proviruses in the latent reservoir increase barrier to HIV-1 cure. Cell. 2013;155(3):540–51. Epub 2013/11/19. doi: 10.1016/j.cell.2013.09.020 ; PubMed Central PMCID: PMC3896327. 24243014 PMC3896327

[9] Dieffenbach CW, Fauci AS. Thirty years of HIV and AIDS: future challenges and opportunities. Ann Intern Med. 2011;154(11):766–71. Epub 2011/06/02. doi: 10.7326/0003-4819-154-11-201106070-00345 . 21628350

[10] Durand CM, Blankson JN, Siliciano RF. Developing strategies for HIV-1 eradication. Trends Immunol. 2012;33(11):554–62. Epub 2012/08/08. doi: 10.1016/j.it.2012.07.001 ; PubMed Central PMCID: PMC3963166. 22867874 PMC3963166

[11] Barouch DH, Deeks SG. Immunologic strategies for HIV-1 remission and eradication. Science. 2014;345(6193):169–74. Epub 2014/07/12. doi: 10.1126/science.1255512 ; PubMed Central PMCID: PMC4096716. 25013067 PMC4096716

[12] El-Sadr WM, Lundgren JD, Neaton JD, Gordin F, Abrams D, Arduino RC, et al. CD4+count-guided interruption of antiretroviral treatment. New Engl J Med. 2006;355(22):2283–96. WOS:000242355300004. doi: 10.1056/NEJMoa062360 17135583

[13] Julg B, Dee L, Ananworanich J, Barouch DH, Bar K, Caskey M, et al. Recommendations for analytical antiretroviral treatment interruptions in HIV research trials-report of a consensus meeting. Lancet Hiv. 2019;6(4):E259–E68. doi: 10.1016/S2352-3018(19)30052-9 WOS:000463017000016. 30885693 PMC6688772

[14] Margolis DM, Deeks SG. How Unavoidable Are Analytical Treatment Interruptions in HIV Cure-Related Studies? J Infect Dis. 2019;220(220 Suppl 1):S24–S6. Epub 2019/07/03. doi: 10.1093/infdis/jiz222 ; PubMed Central PMCID: PMC6636248. 31264691 PMC6636248

[15] Bailon L, Mothe B, Berman L, Brander C. Novel Approaches Towards a Functional Cure of HIV/AIDS (vol 80, pg 859, 2020). Drugs. 2020;80(9):869–. doi: 10.1007/s40265-020-01334-8 WOS:000538516100002. 32495275 PMC7289770

[16] Clarridge KE, Blazkova J, Einkauf K, Petrone M, Refsland EW, Justement JS, et al. Effect of analytical treatment interruption and reinitiation of antiretroviral therapy on HIV reservoirs and immunologic parameters in infected individuals. Plos Pathogens. 2018;14(1). doi: 10.1371/journal.ppat.1006792 WOS:000424003200022. 29324842 PMC5764487

[17] Strongin Z, SharaF R, VanBelzen DJ, Jacobson JM, Connick E, Volberding P, et al. Effect of Short-Term Antiretroviral Therapy Interruption on Levels of Integrated HIV DNA. Journal of Virology. 2018;92(12). doi: 10.1128/JVI.00285-18 WOS:000433416900011. 29593048 PMC5974505

[18] Salantes DB, Zheng Y, Mampe F, Srivastava T, Beg S, Lai J, et al. HIV-1 latent reservoir size and diversity are stable following brief treatment interruption. Journal of Clinical Investigation. 2018;128(7):3102–15. doi: 10.1172/JCI120194 WOS:000437234600040. 29911997 PMC6026010

[19] Calin R, Hamimi C, Lambert-Niclot S, Carcelain G, Bellet J, Assoumou L, et al. Treatment interruption in chronically HIV-infected patients with an ultralow HIV reservoir. Aids. 2016;30(5):761–9. Epub 2016/01/06. doi: 10.1097/QAD.0000000000000987 . 26730568

[20] Pannus P, Rutsaert S, De Wit S, Allard SD, Vanham G, Cole B, et al. Rapid viral rebound after analytical treatment interruption in patients with very small HIV reservoir and minimal on-going viral transcription. J Int AIDS Soc. 2020;23(2):e25453. Epub 2020/02/29. doi: 10.1002/jia2.25453 ; PubMed Central PMCID: PMC7046528. 32107887 PMC7046528

[21] Li JZ, Etemad B, Ahmed H, Aga E, Bosch RJ, Mellors JW, et al. The size of the expressed HIV reservoir predicts timing of viral rebound after treatment interruption. Aids. 2016;30(3):343–53. Epub 2015/11/21. doi: 10.1097/QAD.0000000000000953 ; PubMed Central PMCID: PMC4840470. 26588174 PMC4840470

[22] Williams JP, Hurst J, Stohr W, Robinson N, Brown H, Fisher M, et al. HIV-1 DNA predicts disease progression and post-treatment virological control. Elife. 2014;3:e03821. Epub 2014/09/14. doi: 10.7554/eLife.03821 ; PubMed Central PMCID: PMC4199415. 25217531 PMC4199415

[23] Eriksson S, Graf EH, Dahl V, Strain MC, Yukl SA, Lysenko ES, et al. Comparative analysis of measures of viral reservoirs in HIV-1 eradication studies. PLoS Pathog. 2013;9(2):e1003174. Epub 2013/03/06. doi: 10.1371/journal.ppat.1003174 ; PubMed Central PMCID: PMC3573107. 23459007 PMC3573107

[24] Bruner KM, Murray AJ, Pollack RA, Soliman MG, Laskey SB, Capoferri AA, et al. Defective proviruses rapidly accumulate during acute HIV-1 infection. Nat Med. 2016;22(9):1043–9. Epub 2016/08/09. doi: 10.1038/nm.4156 ; PubMed Central PMCID: PMC5014606. 27500724 PMC5014606

[25] Wang Z, Simonetti FR, Siliciano RF, Laird GM. Measuring replication competent HIV-1: advances and challenges in defining the latent reservoir. Retrovirology. 2018;15(1):21. Epub 2018/02/13. doi: 10.1186/s12977-018-0404-7 ; PubMed Central PMCID: PMC5810003. 29433524 PMC5810003

[26] Fennessey CM, Pinkevych M, Immonen TT, Reynaldi A, Venturi V, Nadella P, et al. Genetically-barcoded SIV facilitates enumeration of rebound variants and estimation of reactivation rates in nonhuman primates following interruption of suppressive antiretroviral therapy. PLOS Pathogens. 2017;13(5):e1006359. doi: 10.1371/journal.ppat.1006359 28472156 PMC5433785

[27] Rosenbloom DIS, Bacchetti P, Stone M, Deng X, Bosch RJ, Richman DD, et al. Assessing intra-lab precision and inter-lab repeatability of outgrowth assays of HIV-1 latent reservoir size. Plos Comput Biol. 2019;15(4):e1006849. Epub 2019/04/13. doi: 10.1371/journal.pcbi.1006849 ; PubMed Central PMCID: PMC6481870. 30978183 PMC6481870

[28] Cohen YZ, Lorenzi JCC, Krassnig L, Barton JP, Burke L, Pai J, et al. Relationship between latent and rebound viruses in a clinical trial of anti-HIV-1 antibody 3BNC117. J Exp Med. 2018;215(9):2311–24. Epub 2018/08/04. doi: 10.1084/jem.20180936 ; PubMed Central PMCID: PMC6122972. 30072495 PMC6122972

[29] Cohn LB, da Silva IT, Valieris R, Huang AS, Lorenzi JCC, Cohen YZ, et al. Clonal CD4(+) T cells in the HIV-1 latent reservoir display a distinct gene profile upon reactivation. Nat Med. 2018;24(5):604–9. Epub 2018/04/25. doi: 10.1038/s41591-018-0017-7 ; PubMed Central PMCID: PMC5972543. 29686423 PMC5972543

[30] Lu CL, Pai JA, Nogueira L, Mendoza P, Gruell H, Oliveira TY, et al. Relationship between intact HIV-1 proviruses in circulating CD4(+) T cells and rebound viruses emerging during treatment interruption. Proc Natl Acad Sci U S A. 2018;115(48):E11341–E8. Epub 2018/11/14. doi: 10.1073/pnas.1813512115 ; PubMed Central PMCID: PMC6275529. 30420517 PMC6275529

[31] Vibholm LK, Lorenzi JCC, Pai JA, Cohen YZ, Oliveira TY, Barton JP, et al. Characterization of Intact Proviruses in Blood and Lymph Node from HIV-Infected Individuals Undergoing Analytical Treatment Interruption. J Virol. 2019;93(8). Epub 2019/02/01. doi: 10.1128/JVI.01920-18 ; PubMed Central PMCID: PMC6450127. 30700598 PMC6450127

[32] De Scheerder MA, Vrancken B, Dellicour S, Schlub T, Lee E, Shao W, et al. HIV Rebound Is Predominantly Fueled by Genetically Identical Viral Expansions from Diverse Reservoirs. Cell Host Microbe. 2019;26(3):347–58 e7. Epub 2019/09/01. doi: 10.1016/j.chom.2019.08.003 . 31471273 PMC11021134

[33] Liu PT, Keele BF, Abbink P, Mercado NB, Liu J, Bondzie EA, et al. Origin of rebound virus in chronically SIV-infected Rhesus monkeys following treatment discontinuation. Nat Commun. 2020;11(1):5412. Epub 2020/10/29. doi: 10.1038/s41467-020-19254-2 . 33110078 PMC7591481

[34] Immonen TT, Camus C, Reid C, Fennessey CM, Del Prete GQ, Davenport MP, et al. Genetically barcoded SIV reveals the emergence of escape mutations in multiple viral lineages during immune escape. Proc Natl Acad Sci U S A. 2020;117(1):494–502. Epub 2019/12/18. doi: 10.1073/pnas.1914967117 ; PubMed Central PMCID: PMC6955354. 31843933 PMC6955354

[35] Khanal S, Fennessey CM, O’Brien SP, Thorpe A, Reid C, Immonen TT, et al. In Vivo Validation of the Viral Barcoding of Simian Immunodeficiency Virus SIVmac239 and the Development of New Barcoded SIV and Subtype B and C Simian-Human Immunodeficiency Viruses. J Virol. 2019;94(1). Epub 2019/10/11. doi: 10.1128/JVI.01420-19 ; PubMed Central PMCID: PMC6912102. 31597757 PMC6912102

[36] Pinkevych M, Fennessey CM, Cromer D, Reid C, Trubey CM, Lifson JD, et al. Predictors of SIV recrudescence following antiretroviral treatment interruption. Elife. 2019;8. Epub 2019/10/28. doi: 10.7554/eLife.49022 ; PubMed Central PMCID: PMC6917497. 31650954 PMC6917497

[37] Pinkevych M, Fennessey CM, Cromer D, Tolstrup M, Sogaard OS, Rasmussen TA, et al. Estimating Initial Viral Levels during Simian Immunodeficiency Virus/Human Immunodeficiency Virus Reactivation from Latency. J Virol. 2018;92(2). Epub 2017/11/10. doi: 10.1128/JVI.01667-17 ; PubMed Central PMCID: PMC5752936. 29118123 PMC5752936

[38] Swanstrom AE, Immonen TT, Oswald K, Pyle C, Thomas JA, Bosche WJ, et al. Antibody-mediated depletion of viral reservoirs is limited in SIV-infected macaques treated early with antiretroviral therapy. J Clin Invest. 2021;131(6). Epub 2021/01/20. doi: 10.1172/JCI142421 ; PubMed Central PMCID: PMC7954603. 33465055 PMC7954603

[39] Deng K, Pertea M, Rongvaux A, Wang L, Durand CM, Ghiaur G, et al. Broad CTL response is required to clear latent HIV-1 due to dominance of escape mutations. Nature. 2015;517(7534):381–5. Epub 2015/01/07. doi: 10.1038/nature14053 ; PubMed Central PMCID: PMC4406054. 25561180 PMC4406054

[40] Wen Y, Bar KJ, Li JZ. Lessons learned from HIV antiretroviral treatment interruption trials. Curr Opin HIV AIDS. 2018;13(5):416–21. Epub 2018/06/08. doi: 10.1097/COH.0000000000000484 . 29878912

[41] Okoye A, Park H, Rohankhedkar M, Coyne-Johnson L, Lum R, Walker JM, et al. Profound CD4+/CCR5+ T cell expansion is induced by CD8+ lymphocyte depletion but does not account for accelerated SIV pathogenesis. J Exp Med. 2009;206(7):1575–88. Epub 2009/06/24. doi: 10.1084/jem.20090356 ; PubMed Central PMCID: PMC2715089. 19546246 PMC2715089

[42] Mueller YM, Do DH, Boyer JD, Kader M, Mattapallil JJ, Lewis MG, et al. CD8+ cell depletion of SHIV89.6P-infected macaques induces CD4+ T cell proliferation that contributes to increased viral loads. J Immunol. 2009;183(8):5006–12. Epub 2009/09/30. doi: 10.4049/jimmunol.0900141 ; PubMed Central PMCID: PMC2757467. 19786539 PMC2757467

[43] Saez-Cirion A, Bacchus C, Hocqueloux L, Avettand-Fenoel V, Girault I, Lecuroux C, et al. Post-Treatment HIV-1 Controllers with a Long-Term Virological Remission after the Interruption of Early Initiated Antiretroviral Therapy ANRS VISCONTI Study. Plos Pathogens. 2013;9(3). doi: 10.1371/journal.ppat.1003211 WOS:000316953800017. 23516360 PMC3597518

[44] Hocqueloux L, Prazuck T, Avettand-Fenoel V, Lafeuillade A, Cardon B, Viard JP, et al. Long-term immunovirologic control following antiretroviral therapy interruption in patients treated at the time of primary HIV-1 infection. Aids. 2010;24(10):1598–601. doi: 10.1097/qad.0b013e32833b61ba WOS:000278636400027. 20549847

[45] Lorenzi JCC, Cohen YZ, Cohn LB, Kreider EF, Barton JP, Learn GH, et al. Paired quantitative and qualitative assessment of the replication-competent HIV-1 reservoir and comparison with integrated proviral DNA. P Natl Acad Sci USA. 2016;113(49):E7908–E16. doi: 10.1073/pnas.1617789113 WOS:000389536700005. 27872306 PMC5150408

[46] Bui JK, Sobolewski MD, Keele BF, Spindler J, Musick A, Wiegand A, et al. Proviruses with identical sequences comprise a large fraction of the replication-competent HIV reservoir. Plos Pathogens. 2017;13(3). doi: 10.1371/journal.ppat.1006283 WOS:000398120300018. 28328934 PMC5378418

[47] Hosmane NN, Kwon KJ, Bruner KM, Capoferri AA, Beg S, Rosenbloom DI, et al. Proliferation of latently infected CD4(+) T cells carrying replication-competent HIV-1: Potential role in latent reservoir dynamics. J Exp Med. 2017;214(4):959–72. Epub 2017/03/28. doi: 10.1084/jem.20170193 ; PubMed Central PMCID: PMC5379987. 28341641 PMC5379987

[48] Maldarelli F, Wu X, Su L, Simonetti FR, Shao W, Hill S, et al. HIV latency. Specific HIV integration sites are linked to clonal expansion and persistence of infected cells. Science. 2014;345(6193):179–83. Epub 2014/06/28. doi: 10.1126/science.1254194 ; PubMed Central PMCID: PMC4262401. 24968937 PMC4262401

[49] Simonetti FR, Sobolewski MD, Fyne E, Shao W, Spindler J, Hattori J, et al. Clonally expanded CD4+ T cells can produce infectious HIV-1 in vivo. Proc Natl Acad Sci U S A. 2016;113(7):1883–8. Epub 2016/02/10. doi: 10.1073/pnas.1522675113 ; PubMed Central PMCID: PMC4763755. 26858442 PMC4763755

[50] Ferris AL, Wells DW, Guo S, Del Prete GQ, Swanstrom AE, Coffin JM, et al. Clonal expansion of SIV-infected cells in macaques on antiretroviral therapy is similar to that of HIV-infected cells in humans. PLoS Pathog. 2019;15(7):e1007869. Epub 2019/07/11. doi: 10.1371/journal.ppat.1007869 ; PubMed Central PMCID: PMC6619828. 31291371 PMC6619828

[51] Montserrat M, Plana M, Guardo AC, Andres C, Climent N, Gallart T, et al. Impact of long-term antiretroviral therapy interruption and resumption on viral reservoir in HIV-1 infected patients. Aids. 2017;31(13):1895–7. doi: 10.1097/QAD.0000000000001560 WOS:000406411400016. 28590333

[52] Long S, Fennessey CM, Newman L, Reid C, O’Brien SP, Li Y, et al. Evaluating the Intactness of Persistent Viral Genomes in Simian Immunodeficiency Virus-Infected Rhesus Macaques after Initiating Antiretroviral Therapy within One Year of Infection. J Virol. 2019;94(1). Epub 2019/10/11. doi: 10.1128/JVI.01308-19 ; PubMed Central PMCID: PMC6912123. 31597776 PMC6912123

[53] Bender AM, Simonetti FR, Kumar MR, Fray EJ, Bruner KM, Timmons AE, et al. The Landscape of Persistent Viral Genomes in ART-Treated SIV, SHIV, and HIV-2 Infections. Cell Host Microbe. 2019;26(1):73–85 e4. Epub 2019/07/12. doi: 10.1016/j.chom.2019.06.005 ; PubMed Central PMCID: PMC6724192. 31295427 PMC6724192

[54] Bruner KM, Wang Z, Simonetti FR, Bender AM, Kwon KJ, Sengupta S, et al. A quantitative approach for measuring the reservoir of latent HIV-1 proviruses. Nature. 2019;566(7742):120–5. Epub 2019/02/01. doi: 10.1038/s41586-019-0898-8 ; PubMed Central PMCID: PMC6447073. 30700913 PMC6447073

[55] Simonetti FR, White JA, Tumiotto C, Ritter KD, Cai M, Gandhi RT, et al. Intact proviral DNA assay analysis of large cohorts of people with HIV provides a benchmark for the frequency and composition of persistent proviral DNA. Proc Natl Acad Sci U S A. 2020;117(31):18692–700. Epub 2020/07/22. doi: 10.1073/pnas.2006816117 ; PubMed Central PMCID: PMC7414172. 32690683 PMC7414172

[56] Kirk GD, Astemborski J, Mehta SH, Ritter KD, Laird GM, Bordi R, et al. Non-structured treatment interruptions are associated with higher HIV reservoir size measured by intact proviral DNA assay in people who inject drugs. J Infect Dis. 2020. Epub 2020/10/11. doi: 10.1093/infdis/jiaa634 . 33037877 PMC8176633

[57] Bertagnolli LN, Varriale J, Sweet S, Brockhurst J, Simonetti FR, White J, et al. Autologous IgG antibodies block outgrowth of a substantial but variable fraction of viruses in the latent reservoir for HIV-1. Proc Natl Acad Sci U S A. 2020;117(50):32066–77. Epub 2020/11/27. doi: 10.1073/pnas.2020617117 ; PubMed Central PMCID: PMC7749285. 33239444 PMC7749285

[58] Pinkevych M, Cromer D, Tolstrup M, Grimm AJ, Cooper DA, Lewin SR, et al. HIV Reactivation from Latency after Treatment Interruption Occurs on Average Every 5–8 Days—Implications for HIV Remission. PLoS Pathog. 2015;11(7):e1005000. Epub 2015/07/03. doi: 10.1371/journal.ppat.1005000 ; PubMed Central PMCID: PMC4489624. 26133551 PMC4489624

[59] Del Prete GQ, Smedley J, Macallister R, Jones GS, Li B, Hattersley J, et al. Short Communication: Comparative Evaluation of Coformulated Injectable Combination Antiretroviral Therapy Regimens in Simian Immunodeficiency Virus-Infected Rhesus Macaques. AIDS Res Hum Retroviruses. 2016;32(2):163–8. Epub 2015/07/08. doi: 10.1089/AID.2015.0130 ; PubMed Central PMCID: PMC4761795. 26150024 PMC4761795

[60] Li H, Wang SY, Kong R, Ding WG, Lee FH, Parker Z, et al. Envelope residue 375 substitutions in simian-human immunodeficiency viruses enhance CD4 binding and replication in rhesus macaques. P Natl Acad Sci USA. 2016;113(24):E3413–E22. doi: 10.1073/pnas.1606636113 WOS:000377948800015. 27247400 PMC4914158

[61] Hansen SG, Piatak M, Ventura AB, Hughes CM, Gilbride RM, Ford JC, et al. Addendum: Immune clearance of highly pathogenic SIV infection. Nature. 2017;547(7661):123–4. Epub 2017/06/22. doi: 10.1038/nature22984 . 28636599

[62] Hansen SG, Piatak M Jr., Ventura AB, Hughes CM, Gilbride RM, Ford JC, et al. Immune clearance of highly pathogenic SIV infection. Nature. 2013;502(7469):100–4. Epub 2013/09/13. doi: 10.1038/nature12519 ; PubMed Central PMCID: PMC3849456. 24025770 PMC3849456

