# Supplementary figures and images for: Transient viral replication during analytical treatment interruptions in SIV infected macaques can alter the rebound-competent viral reservoir
Source: PLoS Pathog. 2021 Jun 18;17(6):e1009686. doi: 10.1371/journal.ppat.1009686 (PMC8244872; doi:10.1371/journal.ppat.1009686)

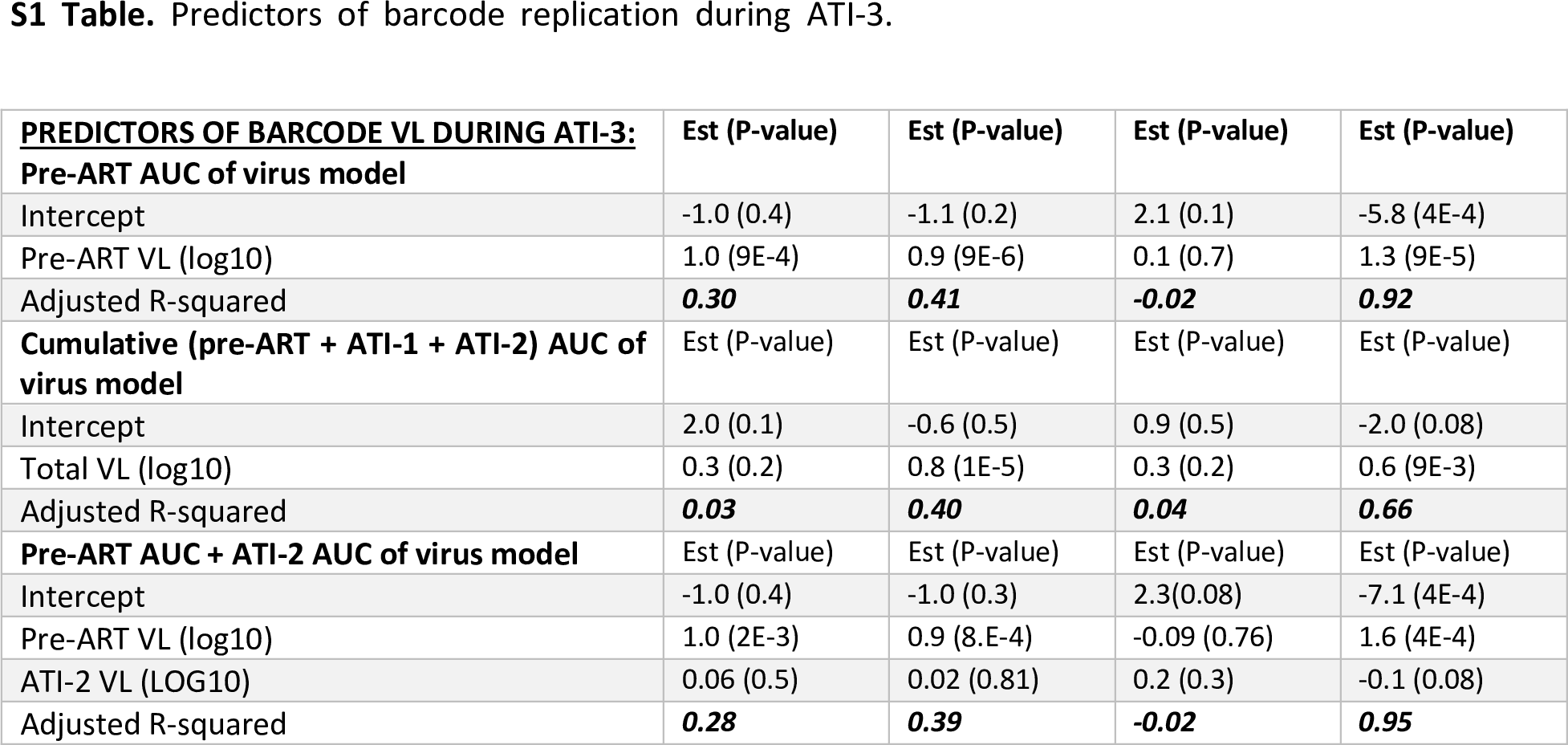

Supplement: S1 Table — Linear regression models for replication of rebounding lineages during ATI-3 based on past replication pre-ART and during ATIs. The sole explanatory variable for the pre-ART model is the pre-ART barcode-specific AUC of virus; the total virus replication model has the sum of barcode-specific AUCs of virus (during pre-ART, ATI-1 and ATI-2) as the explanatory variable. The pre-ART and ATI-2 model includes pre-ART barcode AUC of virus and ATI-2 barcode AUC of virus as independent covariates to allow differential effects of replication during these periods. (TIF) [file ppat.1009686.s001.tif]

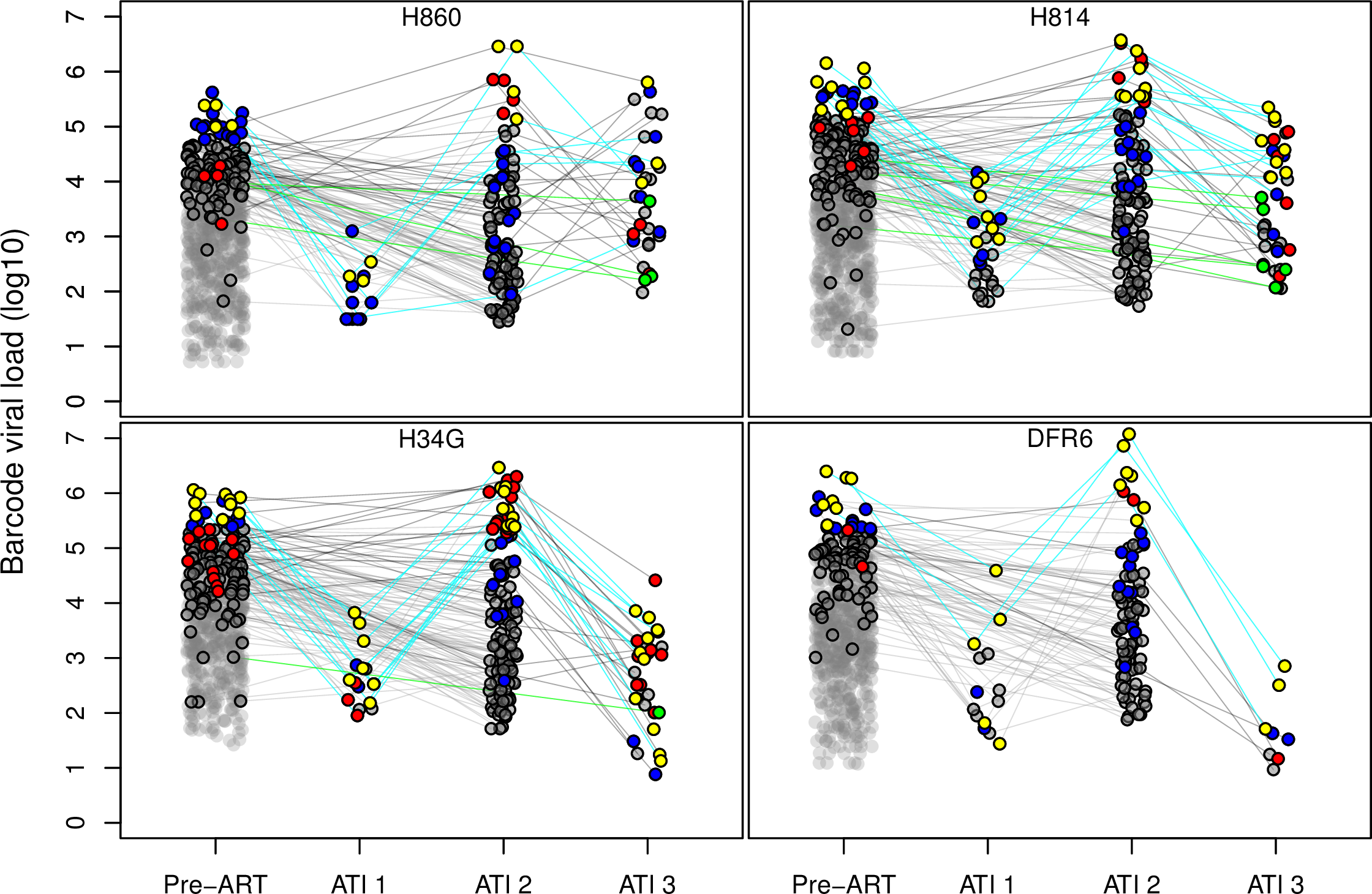

Supplement: S1 Fig — Grey circles indicate viral load (log10) attributable to all barcodes detected in pre-ART plasma. Variants that reactivated during any subsequent ATI are outlined in black. Yellow circles correspond to individual barcodes that were ≥ 1% frequency both pre-ART and during ATI-2, blue circles indicate barcodes ≥ 1% in pre-ART plasma only, red circles indicate barcodes ≥ 1% during ATI-2 only, and green circles indicate barcodes that did not reactivate prior to ATI-3. Cyan lines connect barcodes that were detected across all ATIs while green lines connect barcodes that were only detected in ATI-3. (TIF) [file ppat.1009686.s002.tif]

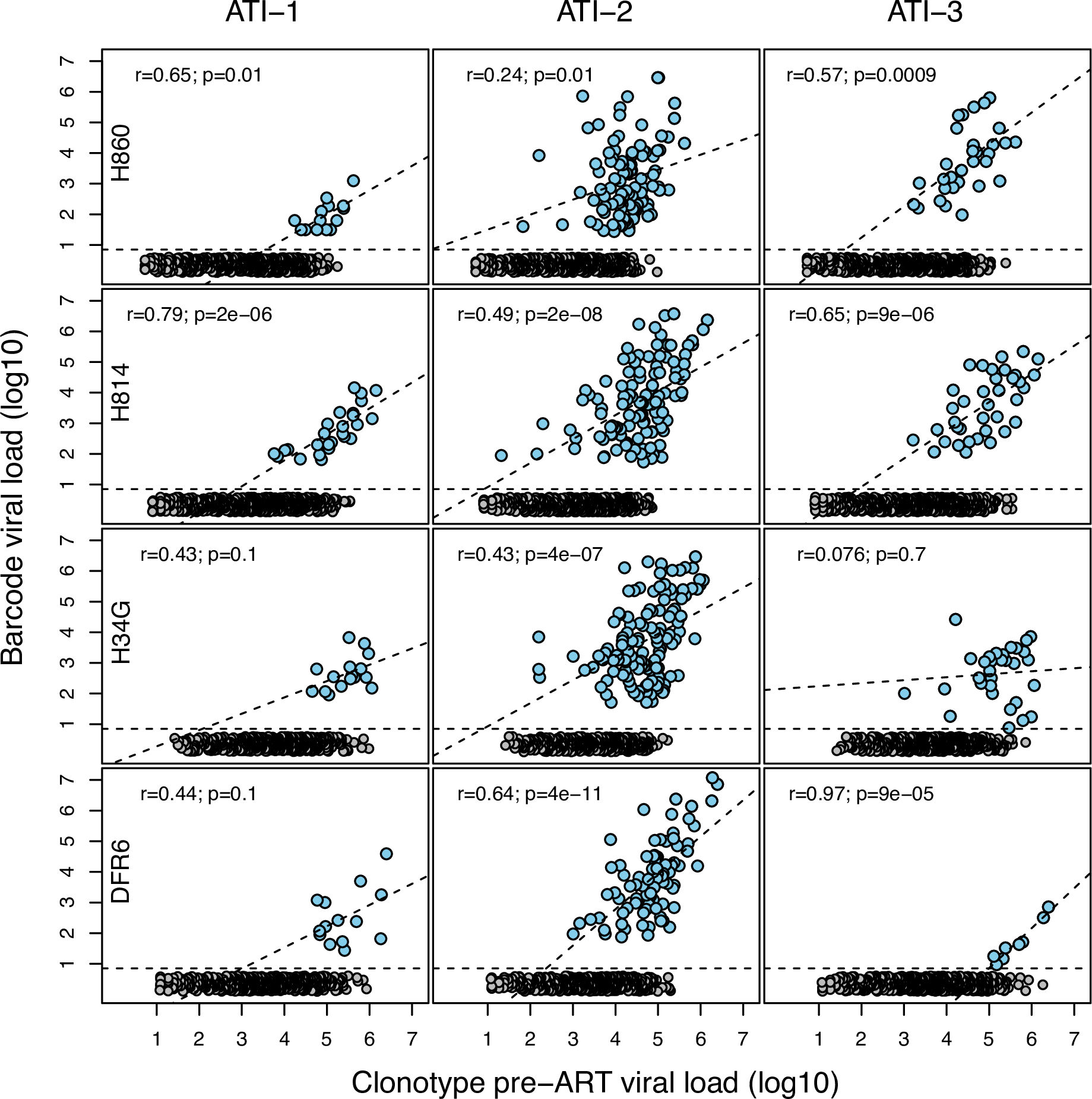

Supplement: S2 Fig — The viral load (log10) attributable to each lineage detected both in pre-ART plasma and during each ATI are shown in blue, while variants not detected during the ATI are shown in grey. The dashed lines depict the linear regression fits. (TIF) [file ppat.1009686.s003.tif]

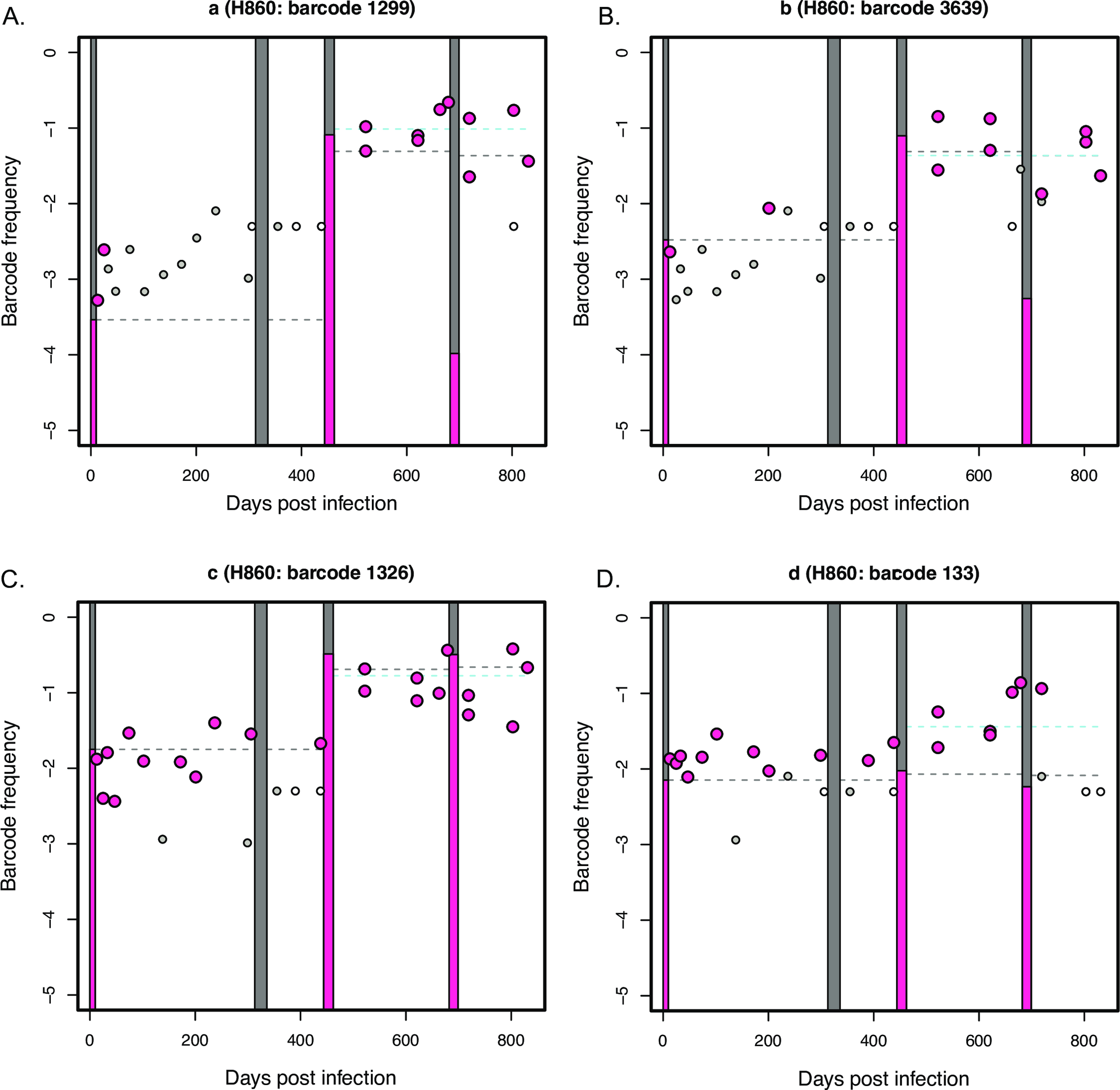

Supplement: S3 Fig — The colored filled circles depict the relative frequencies of the barcodes in viral DNA while the grey circles indicate the limit of detection at time points when a particular variant was not observed. Open circles correspond to the maximum limit of detection for samples where input was not quantifiable. The grey bars highlight the time intervals when the animals were off therapy, with the colored bars indicating the relative frequency of the variant at peak viremia during each interval. The grey dashed lines indicate the relative frequency of each barcode based on cumulative peak plasma viral load. The dashed blue lines indicate the average barcode frequency in PBMC across all post-ATI-2 samples, estimated via maximum likelihood. (TIF) [file ppat.1009686.s004.tif]

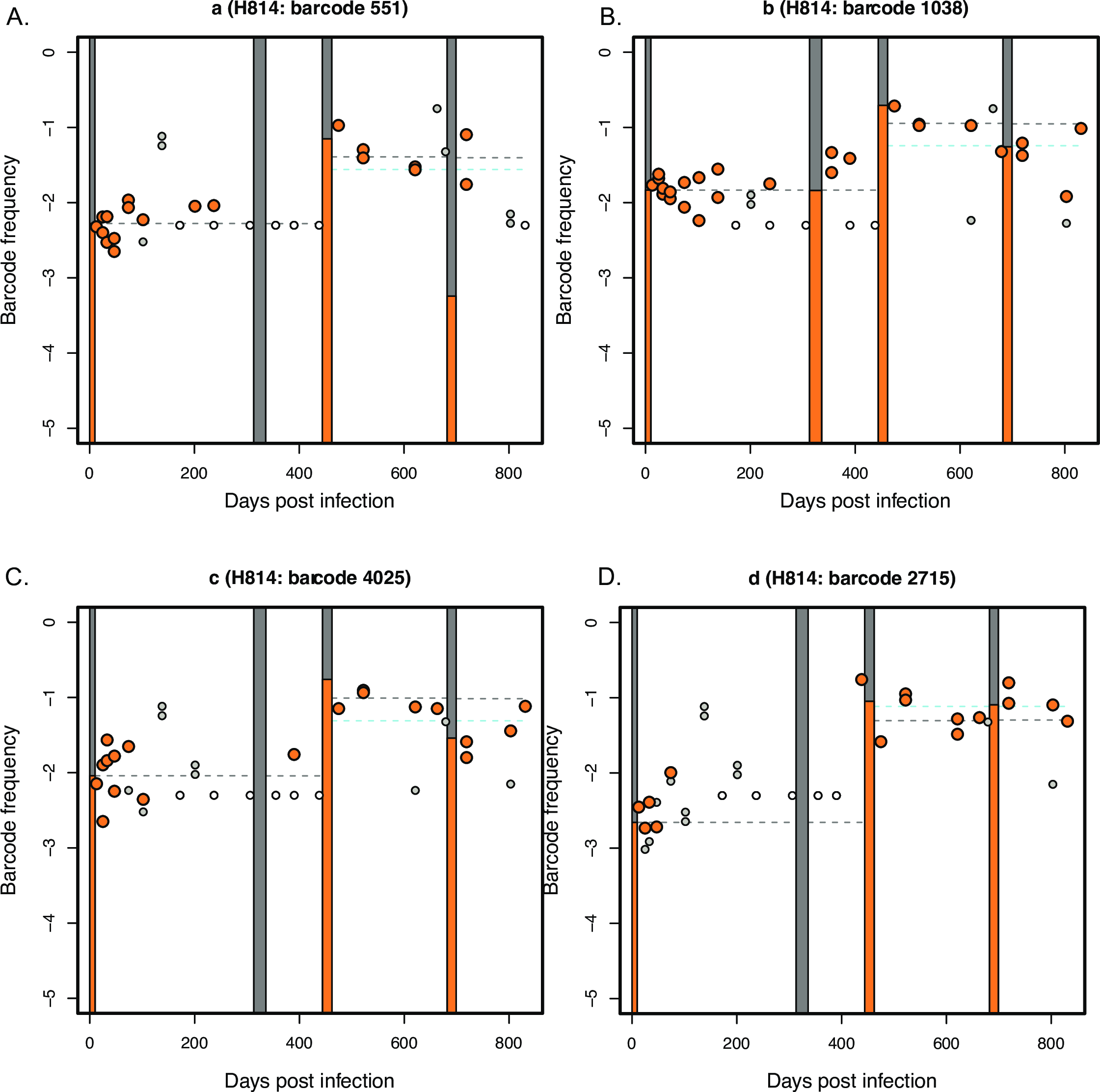

Supplement: S4 Fig — The colored filled circles depict the relative frequencies of the barcodes in vDNA while the grey circles indicate the limit of detection at time points when a particular variant was not observed. Open circles correspond to the maximum limit of detection for samples where input was not quantifiable. The grey bars highlight the time intervals when the animals were off therapy, with the colored bars indicating the relative frequency of the variant at peak viremia during each interval. The grey dashed lines indicate the relative frequency of each barcode based on cumulative peak plasma viral load. The dashed blue lines indicate the average barcode frequency in PBMC across all post-ATI-2 samples, estimated via maximum likelihood. (TIF) [file ppat.1009686.s005.tif]

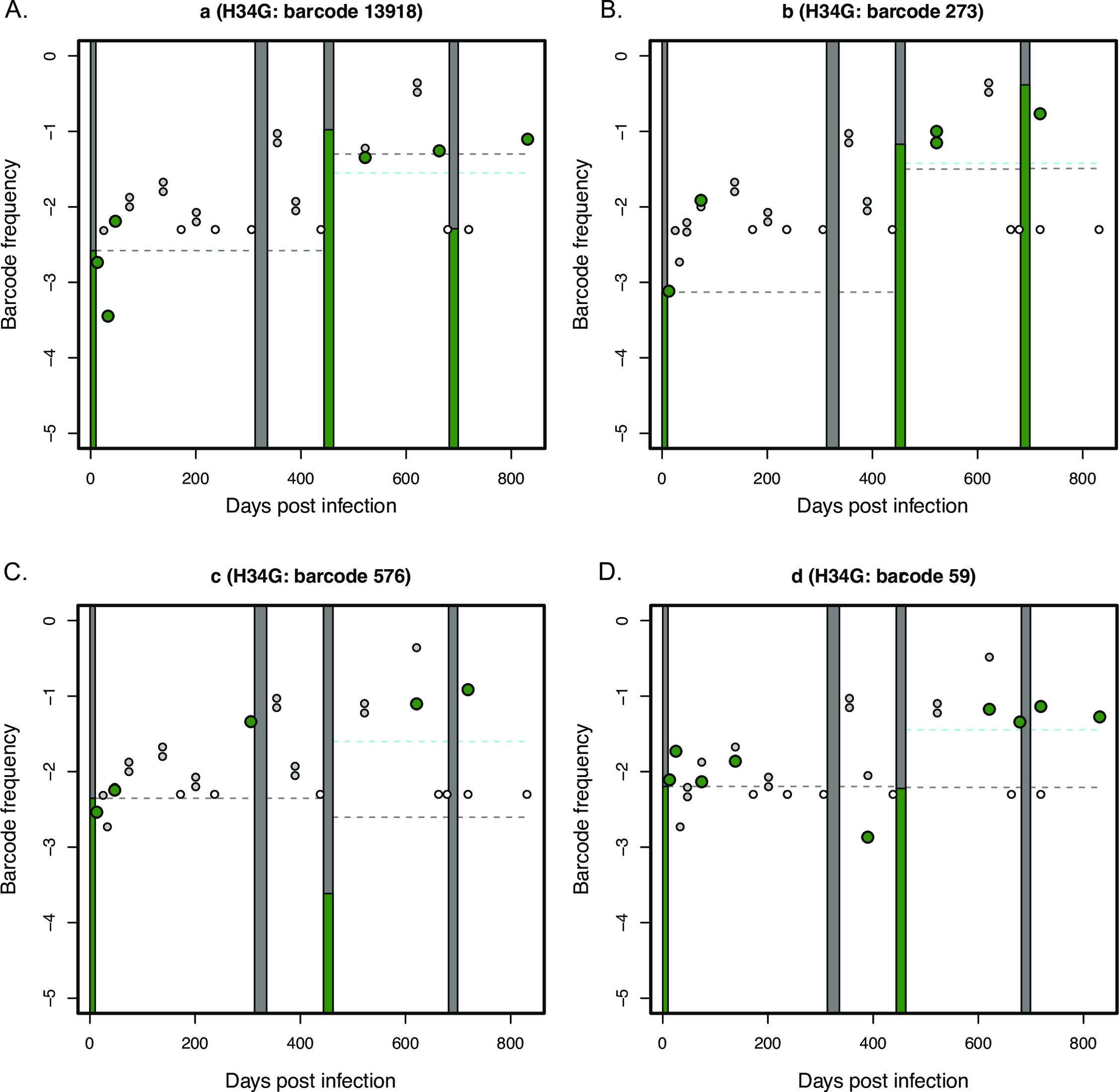

Supplement: S5 Fig — The colored filled circles depict the relative frequencies of the barcodes in vDNA while the grey circles indicate the limit of detection at time points when a particular variant was not observed. Open circles correspond to the maximum limit of detection for samples where input was not quantifiable. The grey bars highlight the time intervals when the animals were off therapy, with the colored bars indicating the relative frequency of the variant at peak viremia during each interval. The grey dashed lines indicate the relative frequency of each barcode based on cumulative peak plasma viral load. The dashed blue lines indicate the average barcode frequency in PBMC across all post-ATI-2 samples, estimated via maximum likelihood. (TIF) [file ppat.1009686.s006.tif]

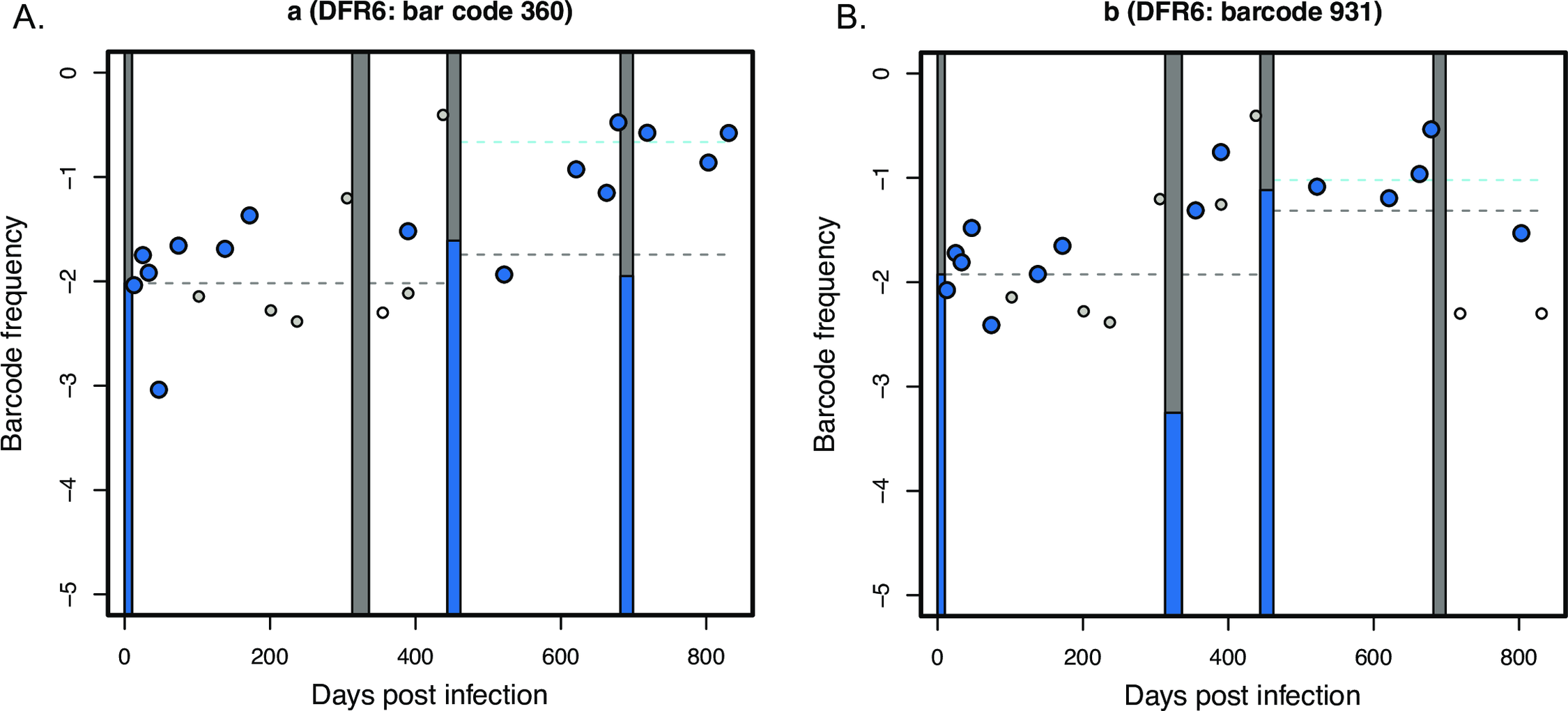

Supplement: S6 Fig — The colored filled circles depict the relative frequencies of the barcodes in vDNA while the grey circles indicate the limit of detection at time points when a particular variant was not observed. Open circles correspond to the maximum limit of detection for samples where input was not quantifiable. The grey bars highlight the time intervals when the animals were off therapy, with the colored bars indicating the relative frequency of the variant at peak viremia during each interval. The grey dashed lines indicate the relative frequency of each barcode based on cumulative peak plasma viral load. The dashed blue lines indicate the average barcode frequency in PBMC across all post-ATI-2 samples, estimated via maximum likelihood. (TIF) [file ppat.1009686.s007.tif]
